# Experimental, theoretical and numerical simulation-based investigations on the fabricated Cu_2_ZnSn thin-film-based Schottky diodes with enhanced electron transport for solar cell

**DOI:** 10.1038/s41598-024-63857-4

**Published:** 2024-07-10

**Authors:** Sachin V. Mukhamale, Moses J. Kartha, Pankaj P. Khirade

**Affiliations:** 1 Department of Physics, Shri Pundlik Maharaj Mahavidyalaya, MS, Nandura Rly, 443404 India; 2 Department of Physics, Savitribai Phule Pune University, Pune, MS 411007 India; 3 Department of Physics, KLE Society’s Science and Commerce College, Kalamboli, Navi Mumbai, MS 410218 India; 4 Department of Physics, Shri Shivaji Science College, Amravati, MS 444603 India

**Keywords:** CZT, Thin film, Electrochemical deposition, Diffusion limitation aggregation, Simulation, Fractal dimension, Materials for devices, Synthesis and processing

## Abstract

Copper-zinc-tin Cu_2_ZnSn (CZT) thin films are promising materials for solar cell applications. This thin film was deposited on a fluorine-doped tin oxide (FTO) using an electrochemical deposition hierarchy. X-ray diffraction of thin-film studies confirms the variation in the structural orientation of CZT on the FTO surface. As the *pH* of the solution is increased, the nature of the CZT thin-film aggregate changes from a fern-like leaf CZT dendrite crystal to a disk pattern. The FE-SEM surface micrograph shows the dendrite fern leaf and sharp edge disks. The 2-D diffusion limitation aggregation under slippery conditions for ternary thin films was performed for the first time. The simulation showed that by changing the diffusing species, the sticking probability was responsible for the *pH*-dependent morphological change. Convincingly, diffusion-limited aggregation (DLA) simulations confirm that the initial structure of copper is responsible for the final structure of the CZT thin films. An experimental simulation with *pH* as a controlled parameter revealed phase transition in CZT thin films. The top and back contact of Ag-CZT thin films based on Schottky behavior give a better electronic mechanism in superstrate and substrate solar cells.

## Introduction

Today’s scenario in the renewable energy science stream has attracted incredible research interest among inventors to develop 2D thin film-based high-efficiency photovoltaic (PV) solar cells^1,2^. Copper-zinc-tin Cu_2_ZnSn (CZT) thin films have the most potential to form quaternary semiconductor compounds with sulfur or selenium (S_x_/Se_1-x_)_4_. These thin films can create a kesterite structure. Kesterite thin films Cu_2_ZnSnS_4_ (CZTS), Cu_2_ZnSnSe_4_ (CZTSe), and its alloys Cu_2_ZnSn(S_*x*_Se_1−*x*_)_4_, (CZTSSe, 0 < *x* < 1), are low cost-effective, with high earth abundant availability, non-toxic, non-vacuum, *p*-type semiconductor, thin-film solar cell material to meet current energy demands, and progress in development of CZTS for solar photovoltaics applications^1,3,4^.

Presently, CZT thin-film technology includes kesterite copper-zinc-tin-sulfur (Cu_2_ZnSnS_4_; CZTS) and chalcopyrite copper-indium-gallium-selenide (Cu(In,Ga)Se_2_; CIGSe), gallium arsenide (GaAs), and cadmium telluride (CdTe). The chalcopyrite thin film-based solar cell has been achieved to have records of efficiencies of 29.1, 22.1, and 23.35%, respectively^5,6^. However, kesterites copper-zinc-tin-sulfide (Cu_2_ZnSnS_4_ or CZTS) and copper-zinc-tin-selenide (Cu_2_ZnSnSe_4_ or CZTSe), and their alloys copper-zinc-tin-sulfide- selenide [(Cu_2_ZnSn(S_*x*_,Se_1−*x*_)_4_ or CZTSSe, 0 < *x* < 1)] have received considerable attention as an absorber layers. Kesterite [Cu_2_ZnSnS_4_, Cu_2_ZnSnSe_4_, and Cu_2_ZnSn(S_x_,Se_1−*x*_)_4_] materials are a potential candidate among CZT thin-film technologies because of their remarkable properties, ideal direct bandgap (1.0–1.5 eV), and high optical absorption coefficient (10^4^–10^5^ cm^−1^) with a crystalline nature^7,8^.

Researchers on kesterite materials have paid scant attention to the need for improved efficiencies since 2005^9^. Various research groups have investigated kesterite materials, and they have reported efficiencies ranging from 5.9 to 12.6% for Cu_2_ZnSn (S_*x*_,Se_1−*x*_)_4_, and 6.77 to 9.4% for Cu_2_ZnSnS_4_ and 9.15 to 11.3% for Cu_2_ZnSnSe_4_, using as an active absorber layer material in superstrate and substrate solar cells^9^. Nevertheless, currently, the kesterite-based solar cell performance is well below the Shockley-Queisser (S-Q) range. The rise in solar cell efficiency has been accompanied by a sharp increase in the prevalence of absorber layer issues in the kesterite structure. Therefore, we aimed to fabricate the CZT thin films, which is imperative because it will lead to CZTS and CZTSe absorber layer for an enhancement of solar cell efficiency and its application. In this study, we investigate whether CZT thin films are related to the growth of the kinetics-based experimental simulation proposed diffusion limited aggregation (DLA) model^10,11^.

In this current work, electrochemical deposition is adopted for the fabrication of CZT thin films with a kesterite structure. Various empirical studies have been conducted on the electrochemical deposition of CZT thin films in absorber layer kesterite structures^12^. These studies have consistently found that the high absorption coefficient and direct band gap of semiconductor CZTS and CZTSe aspects of the absorber layer have the greatest influence on the point-of-contact interface issues, despite this highly enhanced solar cell efficiency, such as CZT thin films which have yet to be robustly researched. Therefore, we focused on the growth mechanisms of CZT thin films, *pH*-dependent phase transition, structural orientation surface morphology, and electron transport mechanisms. While efforts are being made to mitigate these secondary phases in CZT thin films, researchers are reluctant to consider the scale of the problem. The *pH*-dependent phase transition of the CZT thin film approach presented here is radical, but commensurate with the issue.

Kinetic growth of CZT thin films based on 2D Monte-Carlo simulation and its proposed DLA model has attracted considerable research interest for possible device applications^13,14^. CZT thin films are emerging trends in photovoltaic solar cell applications, nanotechnology, and engineering physics. CZT thin films have secondary phases that can affect solar cell performance^15,16^.

These materials can tune size, shape, and geometry to form stable identified phases^17^. Commercial CZT thin films are also employed in optoelectronic devices. This gives a wide exposure to the sustainability of electronic devices. CZT thin films have excellent nano-shaped morphology with different shapes as the *pH* of the solution changes. These types of materials are fabricated using various chemical and physical techniques. It includes electroplating of the metal layer, e-beam evaporation, plasma-enhanced chemical vapor deposition, hot wire chemical vapor deposition method, *R-F* and DC magnetron sputtering, sol-gel spin-coating, pulsed laser deposition, chemical bath deposition, thermal evaporation method, spray pyrolysis, ink coating, and electrochemical deposition, etc.^18,19^. Out of these methods, electrochemical deposition is one of the most common, non-vacuum chemical methods, and is easy to handle and control. Also, it can be deposited in large-scale *p*-type semiconductors on conducting substrates, and is low-cost industrial, and commercial production^20^.

We have optimized the parameter of the electro-depositing potential of CZT thin films using cyclic voltammetry^21^. It can control the material properties and growth of CZT thin films on conducting substrates^22^. The growth mechanisms of CZT thin films show leaf-like dendrites to the sharp-edged disk. The typical *pH*-dependent phase transformation and surface morphology of thin films have been extensively studied by experimentally simulated 2D Monte-Carlo simulations and the proposed DLA model^23^. The structural, morphological, and electrical properties of the fabricated CZT thin films are elucidated. Dendrite fern-like leaves and edge disks without any template and physical method are the most prominent thin films for the formation of binary, ternary, and quaternary semiconductors for solar cells, transistors, and biological sensors. Physicists have focused on this type of morphology and phase transformation of nano shaped CZT thin films because of their wide application in optoelectronic devices and bio-sensing applications. Several computer simulation models such as the Eden model, diffusion-limited aggregation (DLA), and deposition-diffusion aggregation (DDA) have been employed to understand the growth mechanisms in such aggregates^24,25^. These models describe the change in surface morphological patterns observed in various non-equilibrium systems, such as DLA-like, dendrite, nodular, fern, disk, needle, tree-like, dense-branching, compact, spiral, and stingy structures.

In the future, solar technology will mainly focus on kesterite structures for the fabrication of solar cells with semiconducting materials. CZT thin films have excellent nano shaped morphology with different phases such as a one-dimensional nano fern, one-dimensional leaf, dendrite, and one-dimensional rod. Nanowires, nano-disks, and well-defined three-dimensional nanostructures: binary, ternary and quaternary semiconductors. It can also form secondary phases such as metal oxide and sulphide phases such as CuS, CuO, CuSe, ZnO, ZnS, SnS, SnO, SnSe, CuZnO, CuZnS, CuZnSe, ZnSnO, ZnSnS, ZnSnSe, and CuZnSnS (CZTS) or CuZnSnSe^26,27^.

Chemical methods can be developed for the fabrication of CZT thin films and further treatment for semiconductor formation and fabrication of optoelectronic devices. A low-cost and qualitative electrochemical method for depositing thin films is well established. The standard Pourbaix diagram and cyclic voltammetry of CZT are fairly consistent with those of experimentally deposited CZT thin films. Dendrite fern-like leaves and edge disk are found during the formation of binary, ternary, and quaternary semiconductor compounds for the fabrication of solar cells, transistors, and biological sensors. In this work, we have reported experimental simulation studies that show a fractal growth in binary thin films. However, a systematic study of the mechanisms of growth of thin films under different conditions has not yet been conducted. This investigation was undertaken to understand the growth mechanism of ternary CZT thin films via electrochemical methods under different *pH* conditions. 2D diffusion-limited aggregation simulation results are also presented. The present work reports the results of the application of both experimental, theoretical, and simulation model-based crystal growth design mechanisms of CZT thin films to understand the growth of microstructural evolution.

Although CZT thin films have been studied in detail, insufficient attention has been paid to CZT thin films and the interface metal-semiconductor point of contact to enhance the electron transport mechanism of thin films. Hence, the implications of CZT thin films should be explored further. It is generally assumed that CZT thin films are the most suitable for CZTS and CZTSe kesterite structures. However, this article suggests that electrochemically deposited technique, structural orientation toward kesterite structure. A brief explanation of the theoretical growth of the kinetics proposed model, the role of screening effect, surface-morphology, experimental simulation proposed DLA model, sticking-probability and fractal-dimension against *pH*, as well as metal-alloy junction-based point of contact Schottky diode *J-V* and *C-V* characteristics have established a new position in photovoltaics.

## Experimental

### Chemicals

All inorganic and organic chemical compounds, copper sulfate, zinc sulfate, stannous chloride, hydrazine hydrate, hydrochloric acid, and ammonia were of AR grade (Sigma-Aldrich) and were used without further purification or refluxing. Double-distilled water (DDW) was used as the solvent. Acetone, diluted chromic acid, and ethanol were used to clean the substrate.

### Experimental

Initially, we set up a workspace table without any vibration from the surroundings. Computer operated potentiostat (Bio-Logic) interface with the EC-Lab software. A typical electrode that can be used to perform cyclic voltammetry experiments is fabricated. We used transparent conductive oxide (TCO) as an electrode, which is a thin layer of conductive fluorine-doped tin oxide (FTO) on one side of silica glass. TCO makes it easy to deposit CZT thin films on this electrode surface.

**Figure 1:**
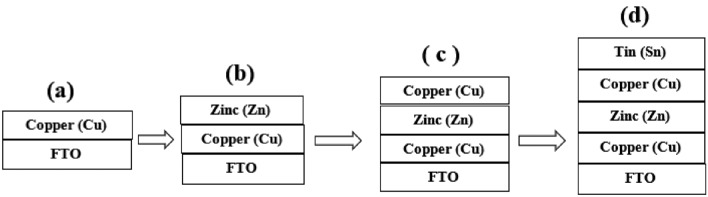
A typical Pourbaix diagram-based block diagram of Cu/Zn/Cu/Sn can be converted to CuZnSn (CZT) thin films at low *pH*, in the range −1 V to +1 V: (**a**) electrochemical deposition of Copper (Cu) on FTO with thickness ~ 32 µm, (**b**) successive electrochemical deposition of zinc (Zn) and copper (Cu) on FTO with thickness ~ 62 µm (**c**) successive electrochemical deposition of copper (Cu) Zinc (Zn) copper (Cu) on FTO with thickness ~ 92 µm, (**d**) successive electrochemical deposition of tin (Sn), copper (Cu), Zinc (Zn) & copper (Cu) on FTO with thickness ~ , (Cu/Zn/Cu/Sn) can be converted to CuZnSn (CZT) thin films.

The electrochemical biological potentiostat (EBS) of the three-electrode cells can be used in our experiments. In the electrochemical deposition, a graphite rod was used as a counter electrode (1.5 × 4.0 cm^2^), a working electrode as a conducting substrate 1.5 × 4.0 cm^2^, a saturated calomel electrode (SCE) was used as the reference electrode, which was connected to the electrochemical biological potentiostat with a buffer solution of double salt bridge system, and an FTO conducting substrate (Sigma-Aldrich) was used as the working electrode. The FTO glass substrates ultrasonically cleaned under acetone, ethyl alcohol, and ultrapure water, each for 15 min. HCl and ammonia were added to maintain the *pH* of the solution for deposition baths to control the surface shapes of the CZT thin films. The electrochemical deposition of CZT thin films was carried out at 27° C in aqueous electrolyte solutions using a depositing potential-based Pourbaix diagram. Field emission scanning electron microscopy (FE-SEM) and X-ray diffractometry (XRD) were used to determine the obtained morphology and phase identification of thin films. Figure 1 shows a typical hierarchy of the Pourbaix diagram. According to the Pourbaix-based block diagram of Cu/Zn/Cu/Sn can be electrochemically deposited on the basis of CuZnSn (CZT) thin films at low *pH* in the range −1 to +1 V. Figure 1a. Electrochemical deposition of copper (Cu) on FTO was carried out with a thickness of ~ 32 µm, which is confirmed through Tally’s step profilometer (TSP), Fig. 1b. The successive electrochemical deposition of zinc (Zn) & copper (Cu) on FTO was observed with a thickness of ~ 62 µm. Figure 1c. Successive electroplating of copper (Cu), zinc (Zn) and copper (Cu) on FTO with thickness ~ 92 µm, Fig. 1d. Successive electrochemical deposition of tin (Sn), copper (Cu), zinc (Zn) and copper (Cu) on FTO with thickness ~ 135 µm, to form CuZnSn (CZT) alloy thin films.

### Electrochemical deposition of the CZT thin films

**Figure 2:**
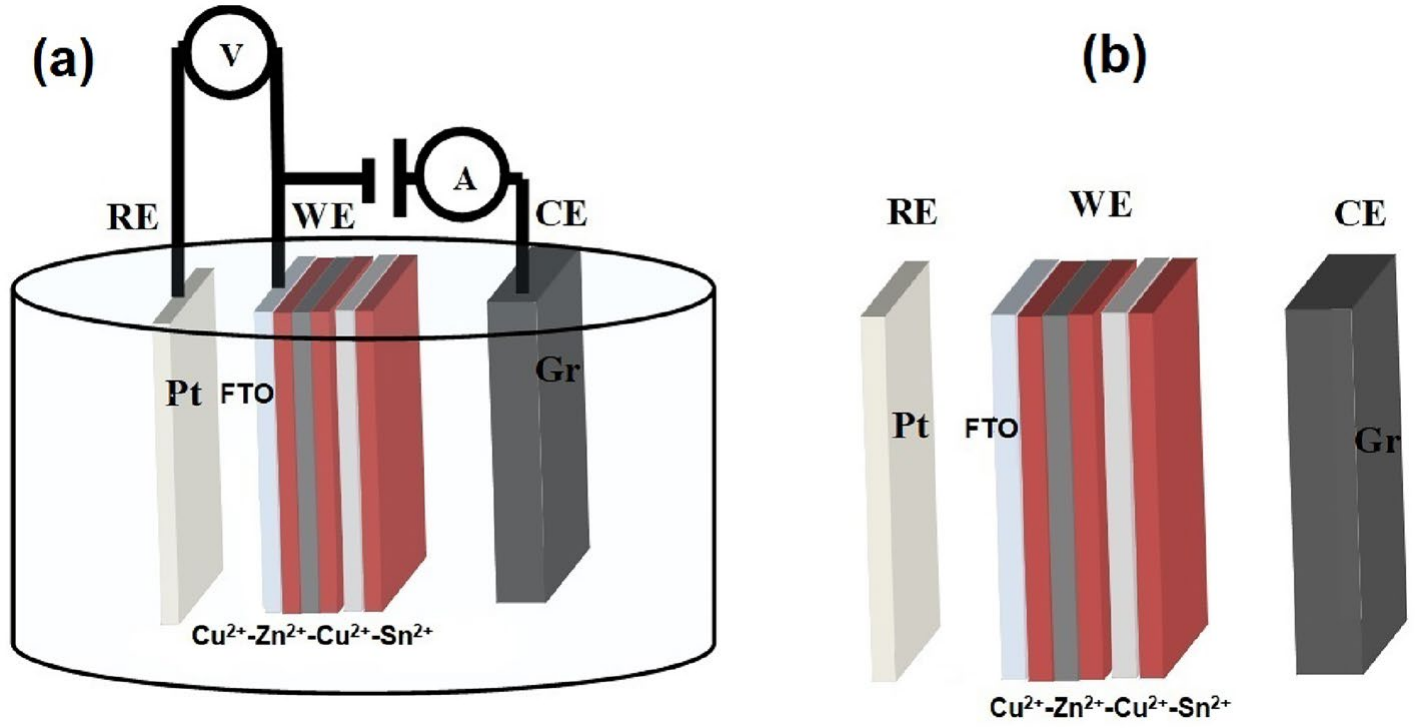
(**a**) The design of electrochemical deposition of multilayer Cu/Zn/Cu/Sn of the successive ionic layer deposited on FTO using reference platinum (Pt) electrode (RE), working as FTO electrode (WE) and counter electrode as graphite (Gr) (CE) connected through an 80 ml transparent electrolyte in 150 ml glass beaker. (**b**) Reference electrode as Platinum, working electrode as FTO and counter electrode are well prepared arrangements with hierarchy of CZT thin films on working electrode.

We performed electrochemical deposition experiments multiple times on FTO substrates. Finally, the established FTO was well cleaned and ultrasonically treated at 25 kHz and 100 W of Toshcon instruments by electroplating copper, zinc, and tin. In the electroplating process, during the film preparation process the time required is 8 h, as in the thin film deposition process, the depositing current is −2.2 to 0.0 mA and its variation during deposition and deposition time is 1–30 min. An analytical chemical reaction was performed to successfully deposit copper-zinc-copper-tin-copper (Cu–Zn–Cu–Sn) on an FTO substrate, as shown in Figs. 2a, b. This is typical of the arrangement of a successive ionic layers of CZT thin films, which can be optimized to obtain a quaternary CZTS semiconductor compound with an annealing sulfur environment with optimized parameters. Figure 1a–d show the hierarchy of CZT thin film formation in terms of a block diagram. The copper precursor contains 0.05 M CuSO_4_ + 0.05 M hydrochloric acid +NH_3_ for *pH*-5, that can be deposited on FTO with a depositing potential at 1.5 V using EBP for 40 min at 27 °C. Similarly, zinc, copper, and tin were deposited by successive ionic layer electroplating as shown in Fig. 2a.

The first step illustrates a uniform and adhesive brown that appears on the FTO substrate as shown in Fig. 2a. The dark gray represents uniform zinc deposited on Cu-FTO in the second step as in Fig. 2b. The blackish gray appears due to Cu–Zn–Cu–FTO deposition in the third step. The silvery white represents the composite of Sn–Cu–Zn–Cu–FTO deposition in the fourth step. The thickness of the successive ionic layer is measured using a talyes-step profile-meter. We found that the growth of thickness increased from 20 to 140 µm and crystallinity of CZT thin films was enhanced after heat treatment.

## Results and discussion

### Theoretical study of the growth mechanism

**Figure 3:**
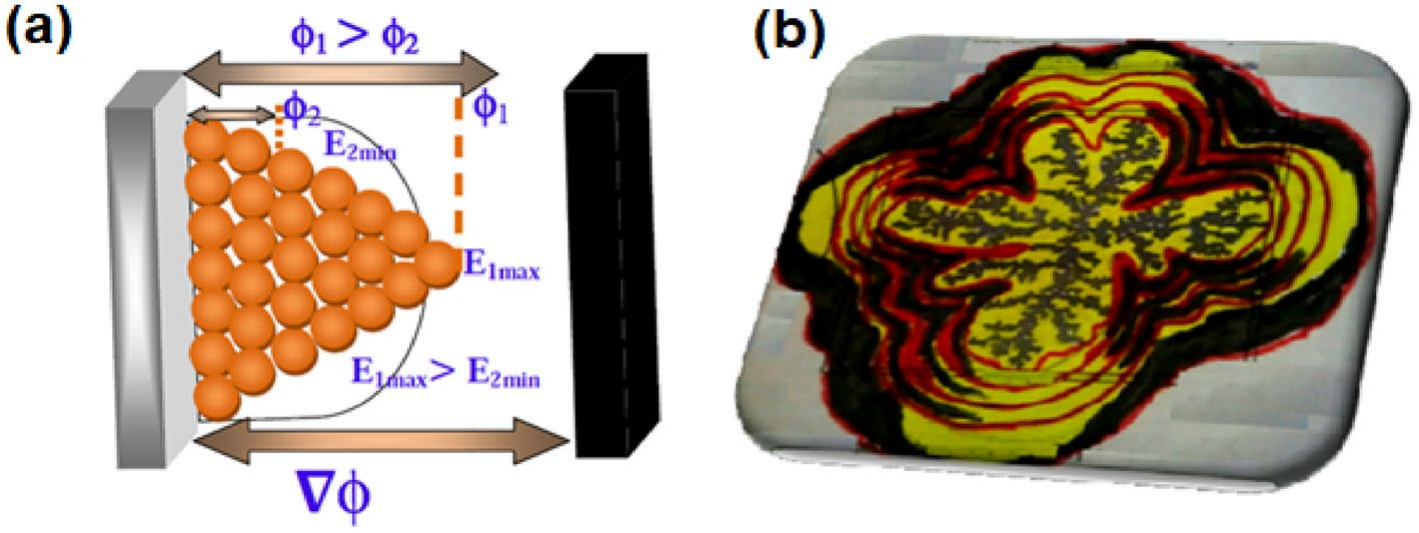
(**a**)The gradient of electric potential across these two electrodes, (**b**) distribution of probability of finding the particle (copper, zinc and tin) randomly diffusing the particle around a diffusion limited aggregate (DLA).

We observed the screening effect, which plays a vital role in a DLA as verified by simulations^28^. Two-dimensional sketch schematic diagrams are shown in Figure 3a, b, respectively. We have proposed a two-dimensional simulation of the diffusion limitation aggregation model. It was observed that maximum ions grow along the interface in two-dimensional DLA. The electric current across the electrode with a constant applied voltage of electro-deposition increases effectively and almost linearly over time, as shown in Fig. 3a, and then the step rises at the early stage of deposit growth. The initial measurements of the electric potential distribution near the interface suggest that diffusion Eq. (1) satisfies the laplace Eq. (2) with boundary condition parameters consistent with experimental and theoretical simulations. Experimental and theoretical simulations carried out using Eqs. (1–7) Ref.^25,29^. Fern leaf-like metal grows almost stationary with diffusion-controlled processes. We consider that the two-dimensional area A of the metal leaf for the transport mechanism of metal ions (ion clusters) is governed by the potential gradient, where *R* is the effective radius of the metal leaf and *t* is the deposition time.

1 $$ \frac{{\partial \phi }}{{\partial t}} = D\frac{{\partial ^{2} \phi }}{{\partial x^{2} }} $$

2 $${\nabla }^{2}\phi =0$$

3 $$\nabla \phi \sim \frac{1}{r}$$

4 $$\frac{dA}{dt}\sim R\nabla \phi $$

5 $$A\sim {R}^{D}$$

6 $$R\sim {t}^{\eta }$$

where, t is the deposition time, $\eta$ is the growth exponent, and

7 $$\eta \sim {D}^{-1}$$

In the case of DLA, a randomly walking particle at a given space and time satisfies Laplace Eq. (2) with the potential *ϕ* replacing with probabilities. Thus, we can find the perimeter growth probabilities by numerically solving the Laplace equation. In the above Eq. (1–7), where, *D* is the diffusion coefficient, *r* is the distance, R is the effective radius of the metal leaf, t is the deposition time and $\eta \sim D^{ - 1}$, $\eta$ is the growth exponent. and *D* is the dimension of the space.

In Fig. 3b, the researchers reported the distribution of the probability of finding the particle (copper, zinc, and tin) randomly diffusing the particle around a diffusion-limited aggregate (dark gray) consisting of ∼25000 particles. This distribution is also analogous to the behavior of the electrostatic potential* ϕ* around a charged conductor having a dark gray cluster. We are exploring the system of equipotential lines depicted using sketch label ink pen instead of the logarithmic scale reported by researchers so that fields drop one order of magnitude between consecutive major layers, demonstrating the screening effect of long fjords.

### Role of the screening effect in ${\varvec{C}}{\varvec{Z}}{\varvec{T}}$ thin films

**Figure 4:**
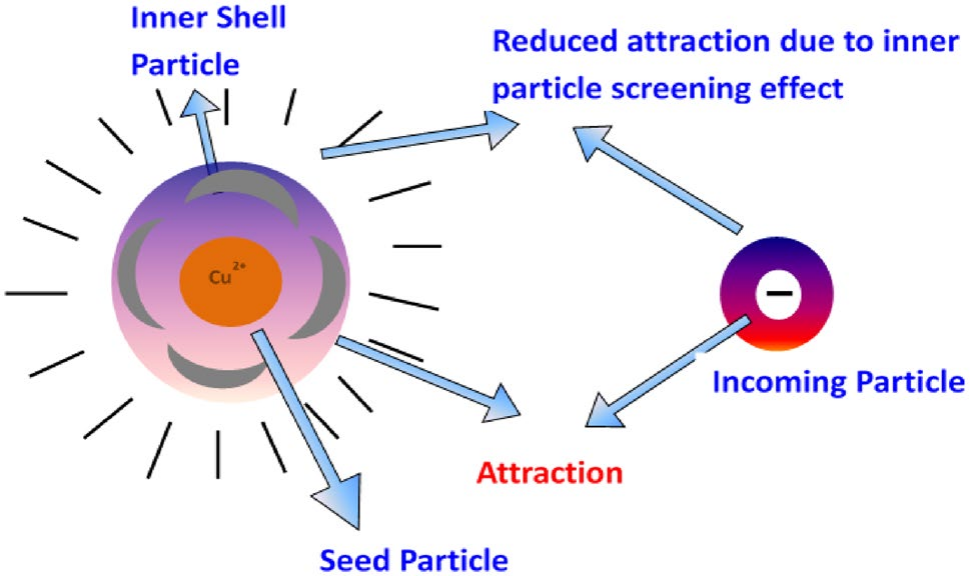
Schematic diagram showing the screening effect in the growth mechanism of CZT thin films, which leads to fractal structures.

Among non-equilibrium models, diffusion-limited aggregation is prominently used to understand the growth mechanism in several physical and biological applications such as viscous fingering, electro-deposition, and bacterial colonies^30^. In the original model, a seed particle is taken in the center of the lattice. For on-lattice simulations, a typical particle is released from a distant point and makes random walks. If the particle visits the neighboring site of the seed, it joins irreversibly with the site. If the distance of the particle crosses a threshold radius; it is excluded from the simulations. Successive particles are considered and they make random walks until they find the cluster neighborhood. The deposited particles screen the penetration of the incoming particle and are there by captured by the outer layer of the aggregates. Thus, the tips in the DLA aggregates grow much faster than the screened part of the cluster. Thus, the density of the cluster decreases, and subsequently, the fractal dimension of the aggregates decreases. The fractal dimensions of 2D DLA aggregates are ~ 1.71 and ~ 2.5 in three dimensions. The screening effects can be reduced by decreasing the sticking probability, which leads to penetration of more particles into the aggregates and fractal dimension increases. In our study, we considered the screening effect by reducing the sticking probability at high *pH,* leading to structural change from fractal to disk aggregates. At high *pH*, less screening effect and high fractal dimensions were obtained in the range of ~ 1.72 to ~ 2.00. A pictorial representation of such screening effects in the growth mechanism of CZT thin films is shown in Fig. 4.

### Structural and compositional analysis

**Figure 5:**
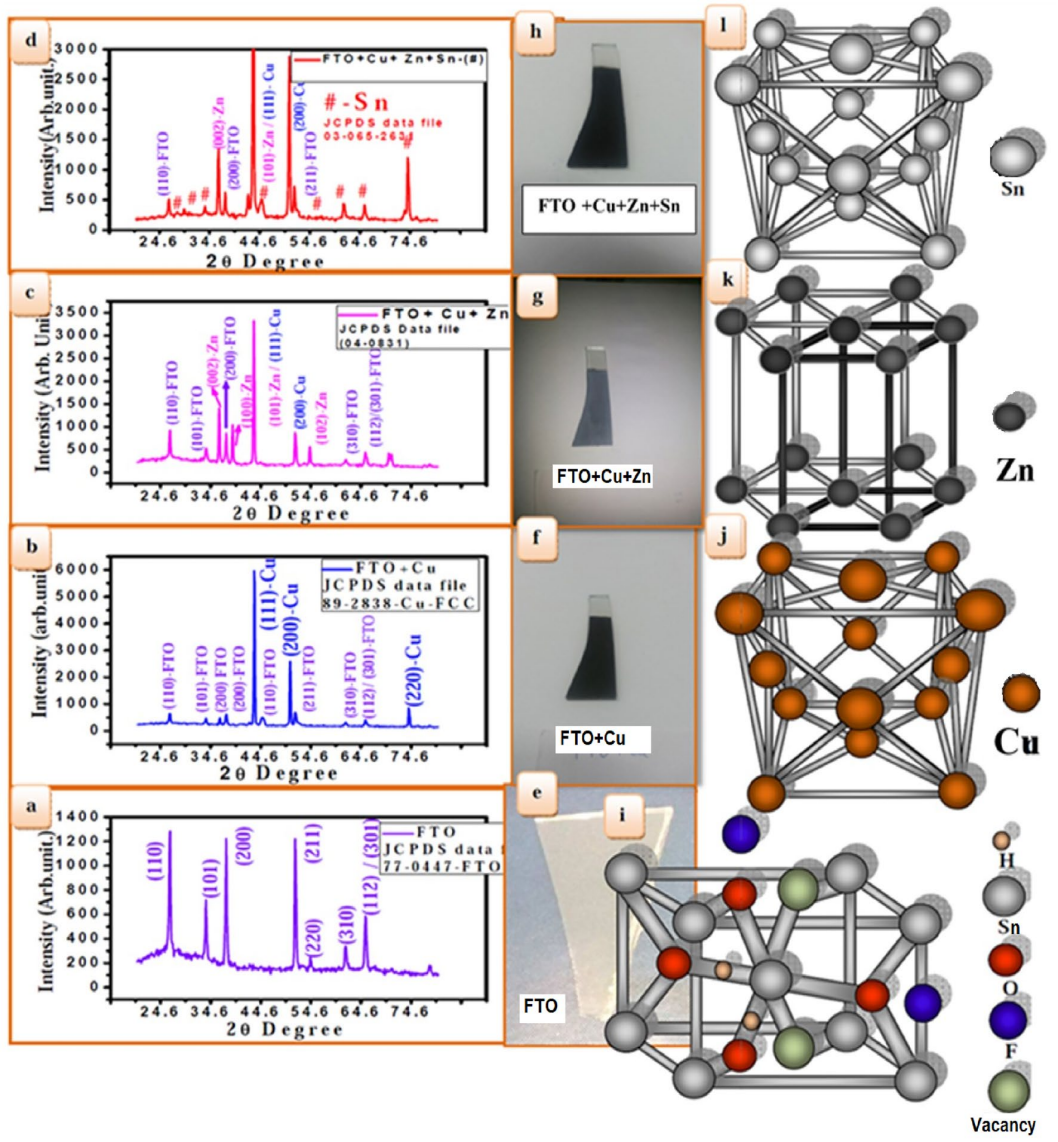
(**a**) XRD pattern of the rutile phase of FTO (**b**) XRD pattern for the electrochemical deposition of copper deposited on FTO (FTO + Cu), (**c**) XRD pattern for the electrochemical deposition of zinc deposited on copper i.e., (FTO + Cu + Zn), (**d**) XRD pattern for electrochemical deposition of Tin (Sn) deposited on Zinc i.e.(FTO + Cu + Zn + Sn). (**e**) Transparent FTO substrate, (**f**) blackish color of electrochemical copper deposited on FTO (FTO + Cu), (**g**) light blackish color of electroplating of Zinc deposited on copper i.e. (FTO + Cu + Zn), and (**h**) dark blackish color of the electrochemical deposition of Tin (Sn) deposits on Zinc, i.e. (FTO + Cu + Zn + Sn), (**i**) rutile structure confirmed through standard JCPDS data 77-0447-FTO, (**j**) Cu-FCC structure confirmed through standard JCPDS data 89-2838 on FTO, (**k**) Zn HCP structure confirmed through standard JCPDS data 04-0831 on FTO, and (**l**) Sn FCP structure confirmed through standard JCPDS data 03-065-263 on FTO.

The copper precursor of 0.05 M CuSO_4_ +0.05 M hydrochloric acid +NH_3_ at *pH*-5, it is deposited on FTO with a depositing potential of 1.5 V using an electrochemical biological potentiostat for 40 min at 27 °C. Similarly, zinc, copper, and tin were deposited by successive ionic layer electroplating. Structural analysis of the CZT thin films was performed using a D-8 Bruker X-ray diffractometer (XRD) with Cu-*Kα*- radiation (0.15406 nm) in the range 2*θ* = 12° to 80°. X-ray diffraction measurements of the CZT thin films are shown in Fig. 5a–d.

XRD spectra of CZT thin films deposited on the FTO substrate, indicating the formation of a chalcopyrite structure. The (112), (103), (110), (204), (220), (116), (312), (200), (105), and (220) Miller indices represent the crystalline phase of CZT, and the characteristic peak (220) represents Sn (Fig. 5d). Originating peaks of (112) and (200) are found in Figs. 5a–c. FTO peaks were also observed in the XRD spectra of CZT materials in Fig. 5a, which is confirmed through rutile structure standard JCPDS #77-0447-FTO, which is constructed in Fig. 5i, the Miller indices of plane consisting of standard JCPDS data of chalcopyrite structure.

Figure 5e is a digital photograph of FTO, (f)-(h) shows the digital photographs obtained from the electrochemical deposition film on the FTO at three different successive ionic layers with the same applied potentials based on the Pourbaix diagram, (f) represents the electrochemical deposition of copper deposited on FTO (FTO + Cu), and (g) shows the deposition electroplating of zinc deposited on copper. (FTO + Cu + Zn), (h) has an electroplating of tin (Sn) deposit on zinc i.e. (FTO + Cu + Zn + Sn), which is depicted as rutile for FTO, face-centered cubic for copper, hexagonal for zinc, and Sn for FCC respectively. Analyses of these structures were confirmed using X-ray diffraction patterns with a standard JCPDS data file. These XRD graphs show the structure with phase identified with a single metal, bi-metal, tri-metal, or thin films, as shown in Fig. 5a–d in a stratified systematic manner. Figure 5e shows the XRD graph of FTO identified with their unique structures such as rutile tetragonal, face-centered cubic (FCC) of Cu appearing on FTO, and hexagonal (HCP) of Zn on (FTO + Cu). It is observed that copper on FTO is a single metal without any cuprite (Cu_2_O) and oxide (CuO) phase, as the main FCC phase, as shown in Fig. 5f. This means that Cu^2+^ ion is totally reduced on the cathodic electrode (FTO) in the electrolyte across the applied potential. The XRD patterns clearly show sharp narrow peaks of (111), (200), and (220) for FCC in Fig. 5b, which is consistent with JCPDS data 89-2838 file. This, atomic structure is observed in Fig. 5j Cu-FCC structure on FTO, of the FCC copper with a = 0.42696 nm. The significant applied cathodic potentials notice that Cu^2+^ ion reduced to copper on FTO forms the FCC structure. Even, H^+^ reduction to H_2_ may be compared with the reduction of Cu^2+^ to form Cu; according to the Pourbaix diagram, the local pH is suitable for the formation of copper. Zinc (Zn): Fig. 5g shows the photograph of the electroplated zinc on copper-assisted FTO with the same applied potential, it is characterized by the XRD method, showing (002), (100), (101), and hexagonal closed packed (HCP) structure as shown in Fig. 5c. This is confirmed through the JCPDS data 04-0831^31^, this atomic a structure is observed in Fig. 5k Zn HCP structure on FTO for hexagonal structure, with *a* = 0.2665 nm, *b* = 0.2665 nm, *c* = 0.4350 nm, *α* = *β* = 90° and *γ* = 120°. Tin (Sn): Fig. 5h shows a photograph of Tin (Sn) deposited on (zinc + copper) assisted FTO, which represents the FCC structure according to the standard JCPDS data 03-065-2631. This atomic structure is observed in Fig. 5l on FTO. The XRD graphs in Figure 5b–d carry minor rutile phase peaks as per JCPDS data 77-0447^32^.

### Morphological study

**Figure 6:**
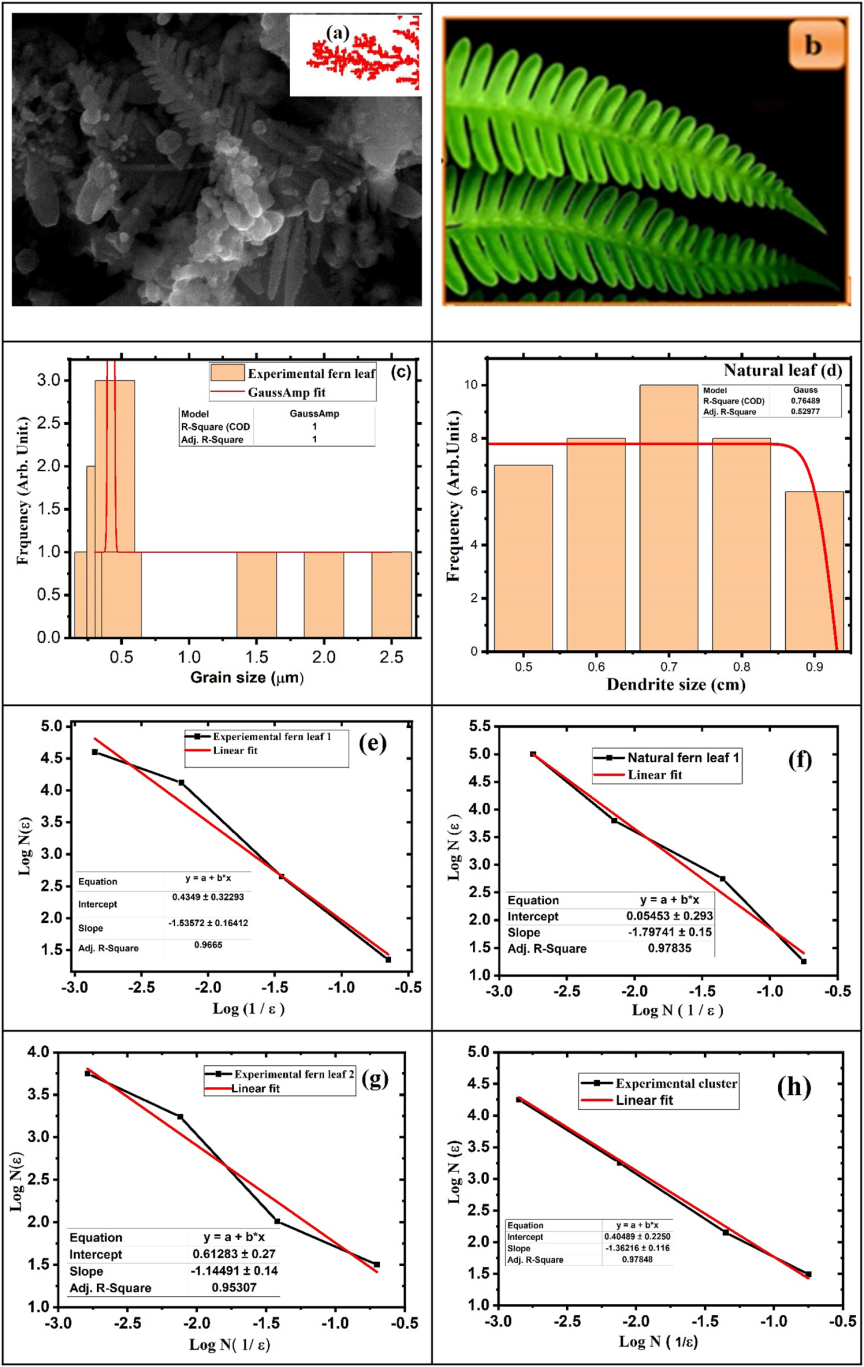
(**a**) Inset of FE-SEM image of CZT dendrite nanostructures fabricated via electrochemical deposition from solutions containing 0.05 M (CuSO_4_ + ZnCl_2_ + SnCl_2_ ) +0.05 M HCl hydrochloric acid at *pH* = 5 for 30 min. (**b**) Natural fern leaf structure shape image obtained from https://images.app.goo.gl/1DxsJg37LaC8xm7FA , (**c**) histogram of experimental fractal-shaped CZT thin film structure, (**d**) histogram of natural fractal-shaped CZT thin film structure, (**e**) log N versus log 1/ε for experimental fern, (**f**) natural fern, (**g**) experimental fern 2, (**h**) experimental cluster.

CZT thin films were characterized by the FE-SEM surface technique. The surface micrograph of the CZT thin-film image shows the fern-like dendrite with clusters. The FE-SEM micrograph clearly shows diffusion limitation aggregation in CZT thin films due to their electroless reduction of copper at typical *pH*, solution concentration, and depositing potential. The Dendrite nanostructure of CZT thin films can diffuse sulfur after sulfurization for a new quaternary semiconductor compound. The reduced copper ion diffuses zinc and tin with screening charges and unstable phases of Cu–Zn, CuOH, and other species. A few clusters appear due to copper hydroxide formation. The dendrite structure of the CZT thin films is similar to that of the fern leaf, as shown in Fig. 6a.

Copper ions play an important role in initiating dendrite fern-like structures. Figure 6a represents natural highly ordered branches that appear in CZT thin films at *pH*-5 for 27 °C. While another structure is due to additional Zn and Sn deposition during electrochemical deposition. This gives the irregular spherical shape of the CZT thin films. The fractal dimension (fd) is calculated using the box counting method by taking the slope from the graph of log N versus log 1/ε as shown in Fig. 6e–h) and in Fig. 6f for experimental fern and natural fern, respectively. The width of the dendrite is around 300–500 nm and the fd is about 1.5–1.7. The length of these leaf-like platelets in the CZT thin film form is ~ 1 µm. The overall length of the CZT dendrite thin film structure is in the range of 6–10 µm depending on the synthetic conditions, and the stem is approximately 500–800 nm in diameter. The leaf-like platelets were well aligned on both sides of the stem. It is clear that the dendrite nanostructure is symmetric, and the angles between the stem and the branches are mostly about 40–50.

We plotted the histogram of natural ferns using Image-J software (version 1.52 a). Figure 6d shows that the left side of a histogram resembles a mirror image of the right side; then, the data are said to be symmetric about Fig. 6b. This means that the natural fern is symmetric to form. It can be observed that the normal distribution of this graph depicts symmetry. The Gaussian curve is exactly a fit to a normal distribution.

Figure 6c shows a histogram of the fractal-shaped CZT thin film structure of particles analyzed by statistical methods. If the dendrite size data are not symmetric, the data are either left-skewed or right-skewed. We observed the positive right skewed for asymmetry and shape of the CZT thin-film structure shown in Fig. 6a, c. The Image-J software gives an average dendrite particle size of 0.25 µm from the histogram plot in Fig. 6c. The Gaussian curve is fitted to the (frequency) number of dendrite particles versus particle size (µm) that gives an exact average size of CZT thin films, dendrite, or fractals. The histogram shows the asymmetry in the mean value of the CZT thin-film particles and the particle size distribution varies from the mean value with a positive (+) skew for varying the microcrystal size. The statistical analysis of image j-software data gives a positive unit value that is closely correlated with SEM, XRD, and Monte Carlo simulations. Mostly, the asymmetry and symmetry structure and shape of CZT thin films required fractal dimension. This fractal dimension gives significance to the self-similarities of the structure. We calculated the fractal dimensions of natural fern and experimental dendrite-like fern with pH-dependent CZT thin films using the box-counting method. The experimental fractal dimension of the CZT thin films fern and fern2 at *pH*-5 was calculated to be approximately ~ 1.17 to ~ 1.7, as shown in Fig. 6e, g. Similarly, the natural fern of the fractal dimension of the CZT thin films is calculated at approximately 1.7 as shown in Fig. 6f. The remaining part of the experimental CZT thin film cluster shows the fractal dimension fd, which is 1.57 as measured from the FE-SEM micrograph and as shown in Fig. 6h.

**Figure 7:**
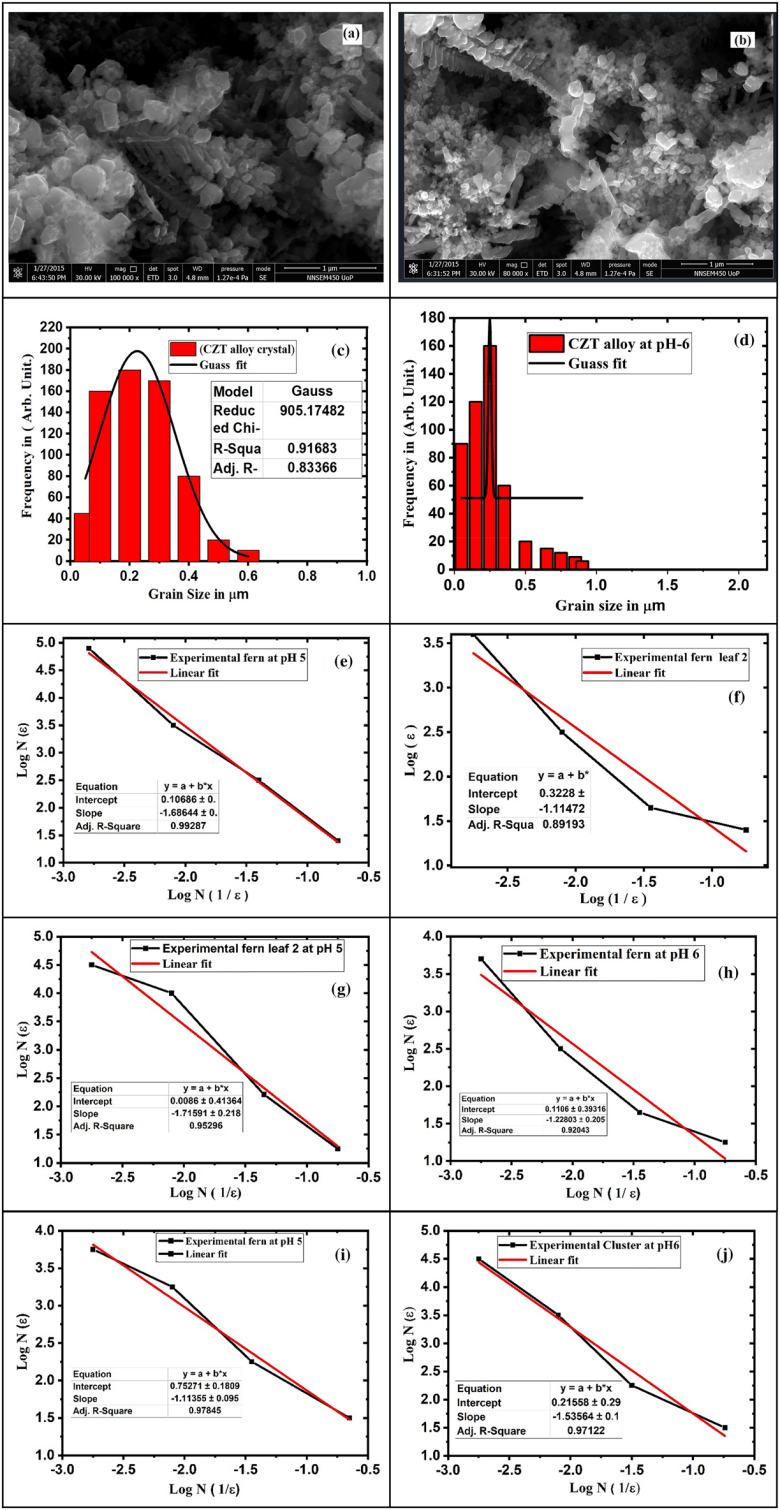
(**a**) Inset of FE-SEM image of CZT dendrite nanostructures fabricated via electrochemical deposition from solutions containing 0.05 M (CuSO_4_ + ZnCl_2_ + SnCl_2_) +0.05 M HCl hydrochloric acid at *pH* = 5 for 30 min. (**b**) for *pH*-6 (**c**) histogram of experimental fractal-shaped CZT thin film structure for *pH*-6 (**d**) histogram of experimental fractal- shaped CZT thin film structure for *pH*-6 (**e**) log N versus log 1/ε for experimental fern at *pH*-5 (**f**) log N versus log 1/ε for experimental fern experimental fern 2 at *pH*-6 (**g**) log N versus log 1/ε for experimental fern 2 at *pH*-5, (**h**) log N versus 1/ε experimental cluster at pH-6(**i**) experimental fern at *pH*-5 (**j**) experimental cluster at *pH*-6.

Figure 7a shows that the CZT thin film morphology has a mixed-phase at constant *pH*-5. The ${Cu}^{2+}$ and ${Zn}^{2+}$ ions are inter-diffused themselves. But ${Sn}^{4+}$ has more reactivity with oxygen for the formation of SnO_2_ and SnO. Cu diffuses in Cu at diffusion coefficient ${D}_{O}\sim 0.20 \times {10}^{-4 }{m}^{2}{s}^{-1}$ and activation energy $\sim 196 kJ {mol}^{-1}$. Zn diffuses in Zn with diffusion coefficient ${D}_{O } \sim 0.15 \times {10}^{-4} {m}^{2}{s}^{-1}$ and its activation energy $\sim 94 kJ {mol}^{-1}$ is responsible for the mixed phase of CZT thin films^33^. DLA has been restricted to control the growth mechanism of CZT thin films. The reactivity of different ionic species depends on the *pH* of the solution. This gives rise to initiating dendrite and other shape morphologies as shown in red dashed circles. Figure 7b represents the maximum nodular shape of the morphology of CZT thin films on the surface of the sample at *pH*-6 upon the addition of NH_3_. A small amount of dendrite growth appears on the surface in Fig. 7b. The ionic concentration of ${Cu}^{2+}$ and ${Zn}^{2+}$ ionic species is less intense and diffuses itself at *pH*-6. CuO, Cu (OH), ZnO, and Zn (OH) are different phases present in the CZT thin films. This metal oxide and metal hydroxide are responsible for the nodular shape in morphology.

Figure 7c shows a right-skewed histogram of a CZT  hin film micrograph at a 1 µm scale for *pH*-5 using Image-J software. This means that CZT thin-film micrographs have an asymmetric form. It is observed that the non-normal distribution of this graph depicts asymmetry. The Gaussian curve is exactly a fit to the non-normal distribution. Figure 7d shows the left-skewed histogram of the CZT thin film micrograph at 1 µm, a scale for *pH*-6, by using the image software. The CZT thin film micrograph shows an asymmetric form structure. The right-skewed histogram of the CZT thin films *pH*-6 of microcrystals (µcs) particles is strongly compressed compared to *pH*-5. It is analyzed using statistical methods. The pH-dependent CZT thin film data are not symmetric, and the data are either left-skewed or right-skewed, representing pH-dependent surface morphology. We observed the positive right skewed for asymmetry and shape of the CZT thin-film structure for *pH*-5 and *pH*-6. The Image-J software gives average particle sizes of 0.24 and 0.22 µm from the histogram plot for *pH*-5 and *pH*-6, respectively.

The Gaussian curve is fitted to the (frequency) of the CZT thin film. The frequency versus particle size (µm) of the film gives the exact average grain size of the CZT thin film, as shown in Figs. 7c, d. The histogram shows the information about the asymmetry in the mean values of the CZT thin films. The particle and particle size distribution varies from the mean value with a positive (+) skew for varying the microcrystal size for *pH*-5 and *pH*-6, respectively. The statistical analysis of image-j software data gives a positive unit value that is closely correlated with SEM, XRD, and Monte-Carlo simulations.

Mostly, the asymmetry and symmetry structure and shape of CZT thin films required fractal dimension. This fractal dimension gives significance to the self-similarities of the structure. We calculated the fractal dimension of *pH*-dependent CZT thin films using the box-counting method. The experimental fractal dimension of CZT thin films from cluster to natural fern at *pH*-5 was calculated to be approximately 1.6 to 1.57 for a 1 µm scale micrograph as shown in Fig. 7e, f. Similarly, the fractal dimension of the CZT thin-film structure from fern to dendrite at pH-6 was calculated at approximately 1.48 to 1.2 for the 1 µm scale of micrograph as shown in Fig. 7g, h. The remaining part of the experimental CZT thin films fern to cluster shows fractal dimension fd, 1.57 to 1.78 is measured from the graph as shown in Fig. 7i, j respectively.

**Figure 8:**
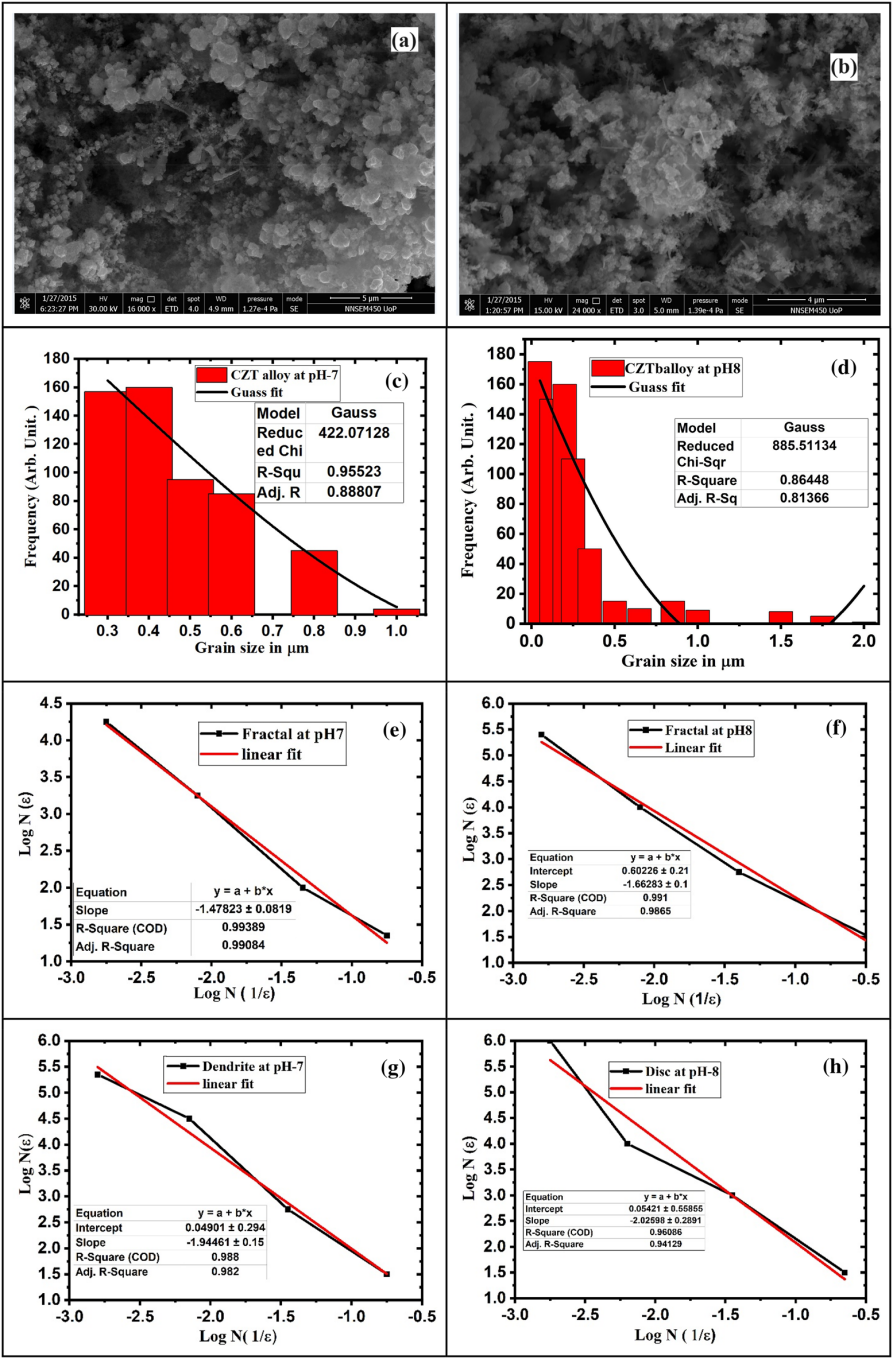
(**a**) FE-SEM image of CZT dendritic nanostructures fabricated via electrochemical deposition from solutions containing 0.05 M (CuSO_4_ + ZnCl_2_ + SnCl_2_) +0.05 M HCl hydrochloric acid at *pH -7* and 27°C for 30 min and (**b**) 0.05 M (CuSO_4_ + ZnCl_2_ + SnCl_2)_ +0.05 M HCl hydrochloric acid at *pH-8* and 27° C for 30 min (**c**) histogram of experimental fractal-shaped CZT thin film structure for *pH-7* (**d**) histogram of experimental fractal-shaped CZT thin film structure for *pH-8* (**e**) log N versus log 1/ε for experimental fern (**f**) experimental fern (**g**) experimental fern 2, (**h**) experimental cluster.

Figure 8a shows that less copper diffusion ionic species with Cu, Zn, and Sn at this *pH-7* for dendrite. Other irregular shape morphologies of CZT thin films have an irrespective diffusion of Cu in Cu, Zn in Zn, and Sn in Sn. Majority of trends in Hydroxide ion formation at the *pH*-7 scale.

Cu(OH) < Zn(OH) < CuO < ZnO < SnO.

The larger irregular shapes of the CZT thin films have overgrowth. This instability on the surface of the film is due to more hydroxide ion diffusion. The successive ionic layers of Cu, Zn, and Sn of the growth mechanisms were found to have various shapes. When pH is increased in firther 8, some of the shapes are totally transformed into the disk of CZT thin films, as shown in Fig. 8b. This type of growth mechanism has been studied by several models like cluster–cluster aggregation, DLA, and the Eden growth model. We observed that Cu is responsible for the dendrite growth of the CZT thin films. DLA was chosen as the most suitable model for the present study^32^.

Figure 8c shows a right-skewed histogram of CZT thin films for granular to dendrite structure of micrograph at 5 µm scale for *pH*-7 using Image-J software. This means that the CZT thin film micrograph shows an asymmetric nature from granular to dendrite microcrystal.

It is observed that the non-normal distribution of this graph depicts asymmetry. The Gaussian curve exactly fits the non-normal distribution. Figure 8d also shows the histogram, which gives information about the right-skewed pattern. This histogram of the CZT thin films is plotted from a micrograph at a 1 µm scale for *pH*-8 using image-J software. The CZT thin film micrograph shows an asymmetric form structure. The right-skewed histogram of CZT thin films for *pH*-8 microcrystals particles is strongly compressed as compared to the *pH*-7. It is analyzed using statistical methods. The *pH*-dependent CZT thin film data are not symmetric, and the data are either left-skewed or right-skewed, representing *pH*-dependent surface morphology. We observed here the positive right skewed for asymmetry and shape of the CZT thin-film structure for *pH*-7 and *pH*-8. The Image-J software gives an average particle size of 0.2 and 0.22 µm from the histogram plot for *pH*-7 and *pH*-8 for granular structure respectively.

The Gaussian curve is fitted to the (frequency) of the CZT thin film. Particles versus particle size (µm) give an exact average grain size of the CZT thin film, as shown in Fig. 8c, d. The histogram shows the asymmetry in the mean value of the CZT thin-film particles and particle size distribution varies from the mean value with a positive (+) skew for varying the microcrystal size for both *pH*-7 and *pH*-8. The statistical analysis of image j-software data gives a positive unit value that is closely correlated with SEM, XRD, and Monte Carlo simulations. Mostly, the asymmetry and symmetry structure and shape of CZT thin films required fractal dimension. This fractal dimension gives the significance of self-similarities of the structure. We calculated fractal dimensions of *pH*-dependent CZT thin films using box-counting method analysis. The experimental fractal dimension of CZT thin films from cluster to natural fern at *pH*-7 was calculated to be approximately 1.4 to 1.9 for 5 µm scale micrographs, as shown in Fig. 8e, g). Similarly, the fractal dimension of the CZT thin-film structure from granular to disk at *pH*-8 was calculated at about 1.87 to 2 for a 4 µm scale of micrograph as shown in Fig. 8f, h.

## Diffusion-limited aggregation and DLA simulation

The experimental study of CZT thin films is discussed here on the basis of the diffusion-limited aggregation (DLA) model and DLA simulation. This statement is proposed to simulate the DLA model on *pH*-dependent phase transition. A two-dimensional Monte-Carlo simulation (MCS) was performed for the generalized diffusion-limited aggregation (DLA) model, which is equivalent to the dielectric breakdown model proposed by Niemeyer et al*.*^33^. The model and experimental simulations reported here provide information about *pH*-dependent phase transitions. Therefore, we can conclude here that this phase transition is observed due to sticking probability against *pH* and fractal dimension against *pH*. A change in the surface morphology of the film has been observed with a change in the *pH* of the solution. Experimentally, morphological change from the dendrite to the disk-like structure is observed in the growth mechanism, however the growth of kinetics involved in this transformation needs to be understood using two-dimensional MCS. Different models have been used to understand the aggregation and growth phenomena. Among these models, the DLA model proposed by Witten and Sander^34^ has been widely used to simulate different systems. DLA models have successfully accounted for structure formation in crystal growth^35^, viscous fingering^36^, biological cells, electro-deposition, and dielectric breakdown^37^. Different variants of the original DLA work have been reported in the literature. This includes slippery ballistic deposition to mimic aggregation of non-shear bonds in a colloidal system, random walkers with drift, DLA aggregation of persistent random walkers, effect of electric field, and the effect of long-range attraction^38^.

**Figure 9:**
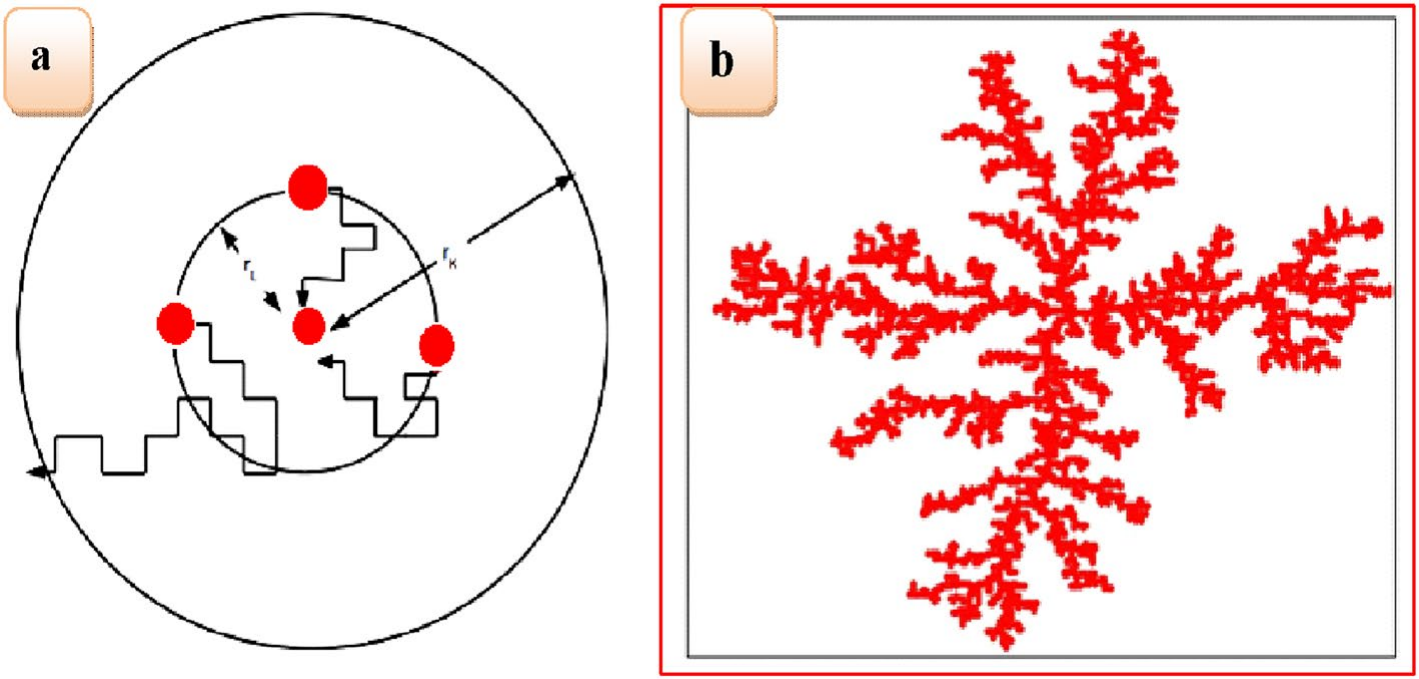
(**a**) Simulation mechanism used in study (**b**) Snapshots of the typical DLA cluster for *p* = 1.

From the XRD and FE-SEM analysis, it is clear that the growth of the kinetics process is dominated by the diffusion mechanism. Therefore, the diffusion-limited aggregation model is the most suitable for understanding the mechanism^39^. In this model, a seed particle is fixed at the center of the lattice. Particles are released in random positions far from the seed from a launching circle of the radius, ${r}_{L}$. The released particle moves following a Brownian trajectory until it reaches one of the four nearest neighbors of the seed, whereupon it sticks, forming a two-particle cluster. Next, we release a new particle that can stick to any of the six perimeter sites of this two-particle cluster. This process is repeatedly iterated. The particle will be killed if the distance from the seed exceeds that of the killing circle radius, ${r}_{K}$ as shown in Fig. 9a. The resulting structure is the result of shadowing generated by branches of the cluster. The slippery DLA analysis revealed the observed morphological changes. The value of sticking probability, *p* is varied from 0.005–1.0 depending on the different conditions. In experiments, we observed that the nature of the film changed from fractal to disk as the *pH* of the solution was increased for *pH* = 5.0 to 8.0. We show that the morphological change is mainly due to an apparent change in the strength of interaction. Therefore, when the value of plies between zero and unity (i.e.; 0 ≤ *p* ≤ 1), it best serves its purpose to simulate such conditions. In classical DLA models, fractal structures are observed for p = 1. However, lowering the value of p close to zero will increase the chances of a particle to diffuse within the branches of fractal structures and for *p* = 1, the original DLA is regained. In this model, the sizes of the individual diffusing particles are considered to be the same. Time is measured, as the number of contacts made between a given diffusing particle and an already deposited particle. The typical DLA scheme employed is shown in Fig. 9a. A typical structure of DLA generated for sticking probability *p* = 1 is shown in Fig. 9a, b. The structures are fractal which is self-similar to ferns in nature, as shown in Fig. 9b.

In the present study, DLA has been considered as the growth mechanism behind the dendrite nature of CZT. An extensive numerical simulation was performed by varying the sticking probability at each stage of growth for different *pH* conditions. The simulation work carried out for the growth of the CZT film depicts the effect of the structure of the copper initially present is responsible for the global structure of CZT thin films.

**Figure 10:**
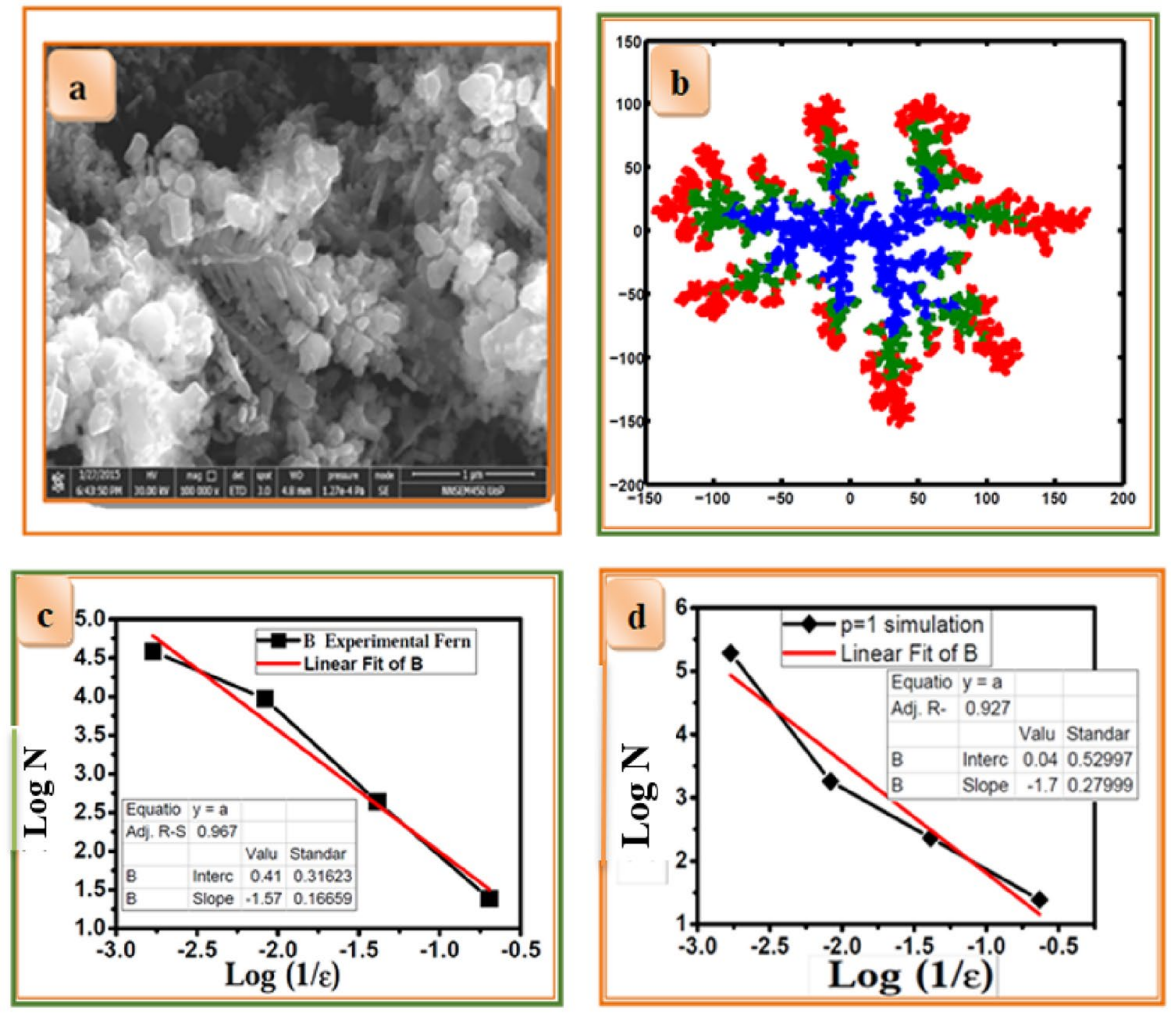
(**a**) FE-SEM image of CZT dendrite nanostructures fabricated via electrochemical deposition from solutions containing 0.05 M (CuSO_4_ + ZnCl_2_ + SnCl_2_) +0.05 M HCl hydrochloric acid at *pH* = 5 for 30 min. (**b**) Typical snapshot of fractal aggregation represents the successive electrochemical deposition of CZT thin films of growth mechanism (**c**) log N versus logN(1/ε) for experimental fern at *pH*-5 (**d**) log N versus log N(1/ε) for simulated fern at *pH*-5.

Schematic 9 (a) shows the growth rule for DLA using Monte-Carlo simulations. Self-similar structures were observed for sticking probability *p* = 1.0. We calculated the fractal dimensions of *pH*-dependent CZT thin films using the box-counting method. The Monte-Carlo simulation depicts the formation of self-similar structures to symmetric structures with increased *pH* of the solution. Figure 10a. FE-SEM micrograph of CZT dendritic nanostructures fabricated via electrochemical deposition from solutions containing 0.05 M (CuSO_4_ + ZnCl_2_ + SnCl_2_) +0.05 M HCl hydrochloric acid at *pH* = 5 for 30 min.

The DLA simulation on a two-dimensional substrate resembles the experimental CZT dendrite nanostructure, as shown in Fig. 10b. The initial part of the simulation is performed with a sticking probability (*p* = 1.0) to mimic copper deposition in experiments. The blue color in Fig. 10b represents the fractal nature of Cu deposition. Similarly, fractal aggregates were observed in an electrolytic copper deposit under diffusion-limited conditions^40^. It provides maximum fractal growth in the material. This results in less diffusion of Cu ions into the material. It is thus proposed that the observed final dendrite nature of CZT nanostructures depends on the initial growth of copper. The fractal growth sustains other ionic elemental species such as Zn and Sn to diffuse consequently.

Further zinc deposition in the experiment is simulated with sticking probability *p* = 0.5, which has a lower sticking probability than the previous plating, as shown in the green region. Zn diffusion in Cu is greater than that of Sn, but aggregations are found more on the surface of Cu. The surface diffusion of Zn in Cu was observed with maximum aggregation. The final deposition of Sn on Cu–Zn is obtained using the same previously applied chemical reaction. The sticking probability of Sn, *p* = 0.25 is very less than that of Cu and Zn. The red region shows the less diffusing ionic species in the Cu and Zn regions. Minimum aggregation of Sn appears in the red region. A typical snapshot of fractal aggregation represents the successive electroplating of CZT thin films by the growth mechanism. The effect of potential, which decays with distance, in addition to the decreased screening effect due to an increase in *pH* was taken into account in the simulation. Thus, for layered deposition, we considered that the diffusion of ions might have increased in each layer, which is modeled as a decreasing sticking probability for Cu, Zn, and Sn.

We compared experimental and simulated fractal dimensions of about 1.57 and 1.7, respectively, for *pH*-5, as shown in Fig. 10c, d respectively. It is observed that the experimental fractal dimension is very close to the standard fractal dimension of the simulated study. The diffusion-limited aggregation of two-component systems with varying sticking probability was studied. It has been shown that for small values of sticking probability, dense structures evolved within the system^41^.

**Figure 11:**
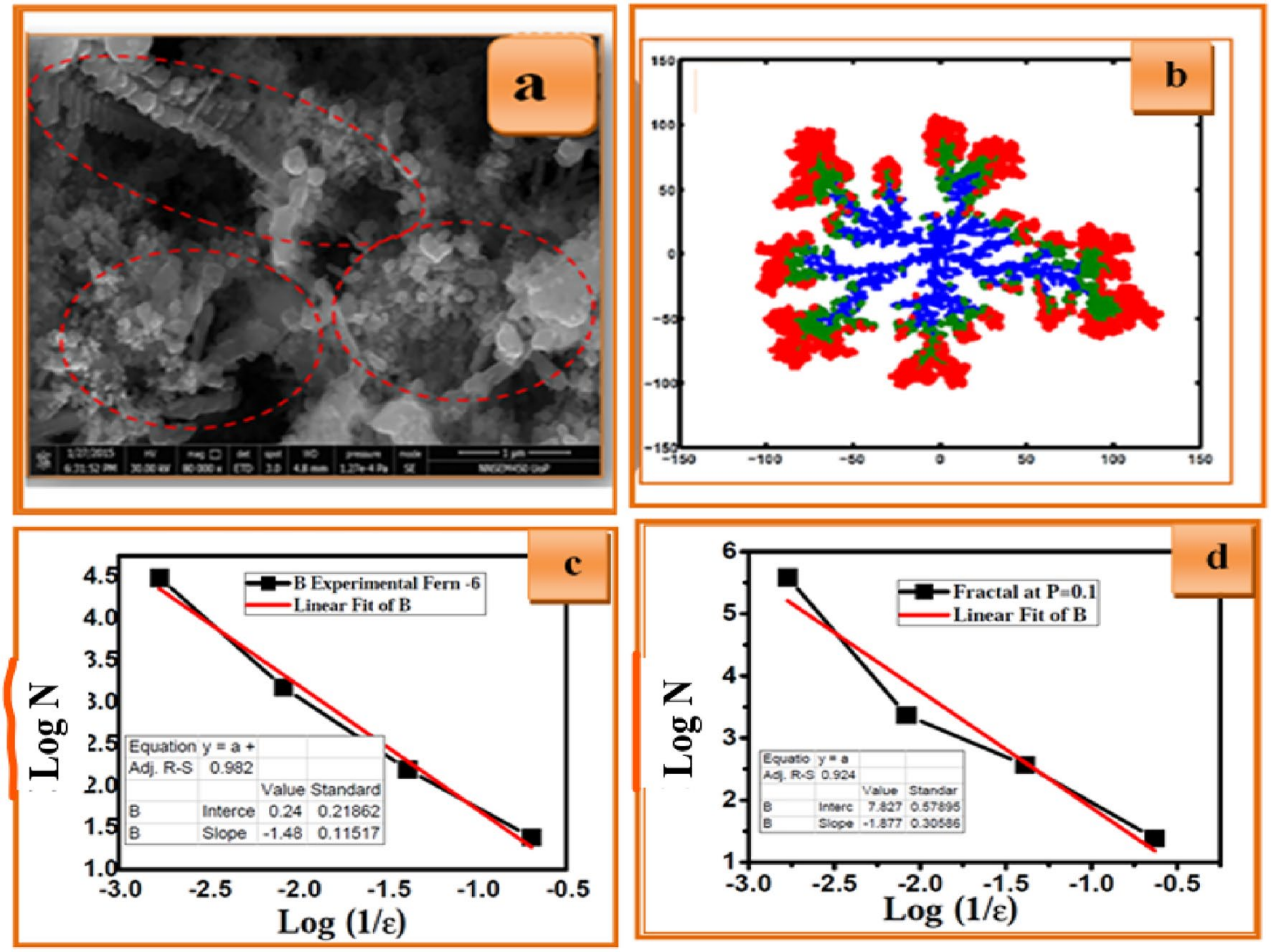
(**a**) FE-SEM image of CZT dendrite nanostructures fabricated via electrochemical deposition from solutions containing 0.05 M (CuSO_4_ + ZnCl_2_ + SnCl_2_) +0.05 M HCl hydrochloric acid at *pH* = 6 for 30 min. (**b**) Typical snapshot of fractal aggregation represents the successive electroplating of CZT thin films of growth mechanism (**c**) log N versus log (1/ε) for experimental fern for *pH*-6 (**d**) log N versus log (1/ε) for simulated fern for *pH*-6.

Figure 11a depicts the FE-SEM micrograph of CZT dendrite nanostructures fabricated via electrochemical deposition from solutions containing 0.05 M (CuSO_4_ + ZnCl_2_ + SnCl_2_) +0.05 M HCl hydrochloric acid at *pH* = 6 for 30 min. DLA simulation on a two-dimensional substrate that resembles the experimental CZT dendrite nanostructure is shown in Fig. 11b. Here, we assume that for lower pH values, the deposition of Cu ions will not be affected. The initial part of the simulation is performed with sticking probability (*p* = 1.0) to mimic copper deposition in experiments. The blue color in Fig. 11b represents the blue fractal nature of the Cu deposition. It provides maximum fractal growth in the material. The fractal growth sustains other ionic elemental species such as Zn and Sn to diffuse consequently.

Further, zinc electrochemical deposition in the experiment was simulated with a sticking probability *p* = 0.1, which has lower sticking probability than the previous plating, as shown in the green region. Zn diffusion in Cu is greater than that of Sn, but aggregations are found more on the surface of Cu. The surface diffusion of Zn in Cu was observed with maximum aggregation. Final deposition of Sn on Cu–Zn obtained from the same previous applied chemical reaction. The sticking probability of Sn *p* = 0.05 is very less than that of Cu and Zn. The red region shows the less diffusing ionic species in the Cu and Zn regions. Minimum aggregation of Sn appears in the red region. A typical snapshot of fractal aggregation represents the successive electroplating of CZT thin films of growth mechanism.

Here, we compared experimental and simulated fractal dimensions of about 1.48 and 1.87 for *pH*-6, as shown in Fig . 11c, d respectively. The experimental fractal dimension is very close to the standard fractal dimension of the simulated study. When *pH* is increased, the screening effects of the depositing particle decrease, which leads to a slightly dense morphology for *pH*-6 compared with the dendritic morphology for *pH*-5.

**Figure 12:**
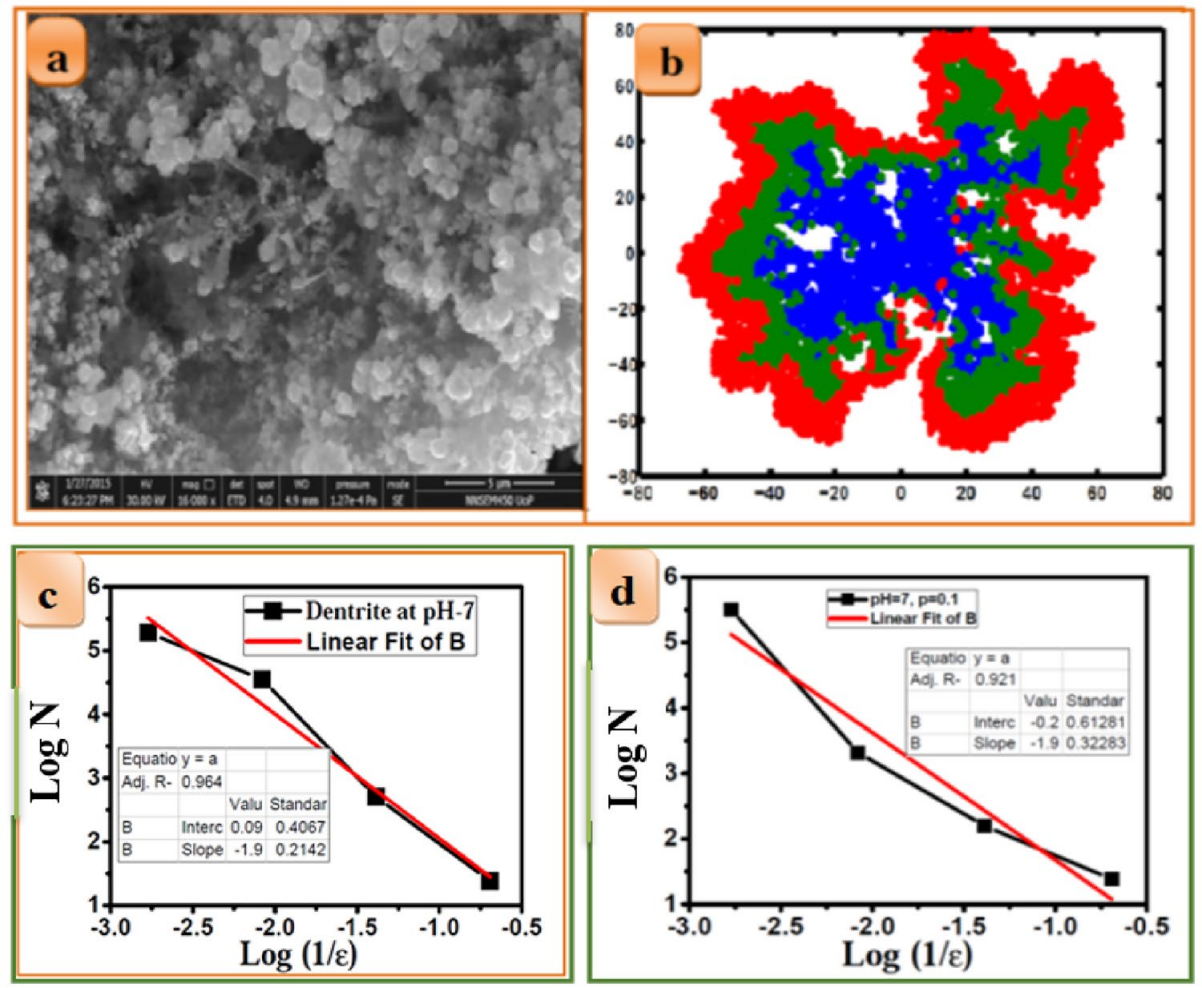
(**a**) 0.05 M (CuSO_4_ + ZnCl_2_ + SnCl_2_) +0.05 M HCl hydrochloric acid +NH_3_ at *pH*-7 and 27^0^ C for 30 min. (**b**) Typical snapshot of fractal aggregation representing the successive electrochemical deposition of CZT thin films of growth mechanism (**c**) log N versus log (1/ε) for experimental fern for *pH* 7 (**d**) log N versus log (1/ε) for simulated fern for *pH* 7.

We can understand the morphology of the cluster due to the change in the *pH* of the solution. Experiments were conducted by increasing the *pH* of the solution. The solution was prepared at *pH* 7 for this molar concentration [0.05 M (CuSO_4_ + ZnCl_2_ + SnCl_2_) +0.05 M HCl hydrochloric acid +NH_3_]. The nodular surface micrograph of the CZT thin films is shown in Fig. 12a. It is clear from the figure that the fractal nature of the aggregates shown in Fig. 12a has changed to a compact structure. Simulation has been performed for Cu with a sticking probability (*p* = 0.1). The lower value of sticking probability was chosen here due to the higher *pH*-7 and 27° C and for 30 min as per standard and practical Pourbaix diagram of electrochemical deposition parameters. The tip of the Cu ions does not grow as rapidly as high *pH* ions due to the screening effect of Cu(OH). The internal self-diffusion of Cu ions in the Cu structure is found to be small at *pH*-7, The Cu(OH) in copper diffusion stops the growth of Cu in Cu diffusion. Hydroxide ionic species are gathered at the end of the wing causing maximum aggregates. Therefore, maximum aggregation causes less DLA with the minor fractal structure shown in the blue region in Fig. 12b. This supports the formation of nodular and granular structures.

The second layer of Zn has a sticking probability *p* = 0.01 is less than the first layer of Cu at the same electrochemical deposition parameter. The diffusion coefficient of Zn here is considered small compared to Cu. The surface diffusion and diffusion coefficient of Zn in Cu are small compared to those of Zn(OH) and ZnO, as per the Pourbaix diagram. The second electroplating shows the wide width of the wing and the end of the tip has more aggregation due to the screening effect of Zn(OH) and ZnO. Therefore, the second electroplating has a less fractal structure at *pH* 6 and 27 °C for 30 min than the previous *pH*-5 case shown in the green region in Fig. 12b. The interfacing diffusion of the first and second electroplating is in the middle of the first blue region, as shown in Fig. 12b. The third layer of Sn electroplating on Cu–Zn has a sticking probability of *p* = 0.005 for Sn. Sn has less sticking probability due to the higher *pH* -7 and 27 °C for 30 min as per standard and practical Pourbaix diagram as compared with previous plating as shown in the blue and green regions, respectively, at the same electrochemical parameter. Sn surface diffusion in Zn is less than the interfacing of Zn–Cu thin films. The tip of the Sn ions does not grow as rapidly as high *pH* ions due to the screening effect of Sn(OH) and SnO. The internal diffusion of Sn in Sn is less at *pH*-6. Sn(OH) in zinc diffuses to stop the growth of Sn. The formation of secondary phases such as Cu (OH), CuO, ZnO, Zn (OH), SnO, and SnO_2_ is observed in the XRD diffraction pattern. Hydroxide ionic species are gathered at the end of the wing causing maximum aggregates. Therefore, maximum aggregation causes less DLA with the minor fractal structure shown in the red region in Fig. 12b. This structure is growing to form a nodular and granular-like structure of CZT thin films as confirmed by the XRD pattern. This is consistent with the FE-SEM micrograph 1 µm scale.

We compared experimental and simulated fractal dimensions of approximately 1.9 and 1.9 for *pH*-6, as shown in Fig. 12c, d) respectively. The *pH*-dependent asymmetry of the experimental fractal dimension is very close to the standard fractal dimension of the simulated study.

**Figure 13:**
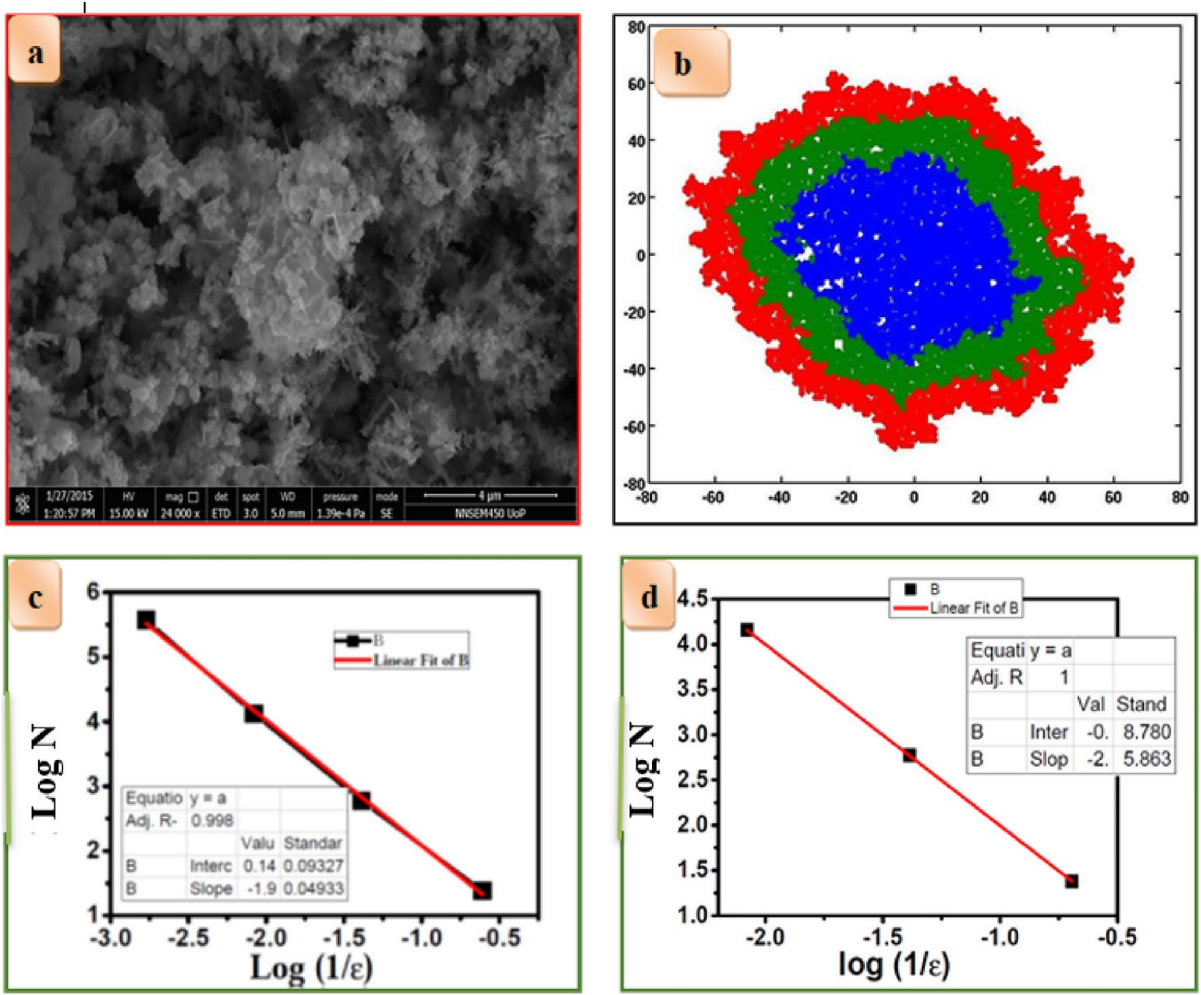
(**a**) 0.05 M (CuSO_4_ + ZnCl_2_ + SnCl_2_) +0.05 M HCl hydrochloric acid at *pH* 8 and 27° C for 30 min. (**b**) Typical snapshot of fractal aggregation represents the successive electroplating of CZT thin films of growth mechanism (**c**) log N versus log (1/ε) for experimental fern for *pH* 8 (**d**) log N versus log (1/ε) for simulated fern for *pH* 8.

0.05 M (CuSO_4_ + ZnCl_2_ + SnCl_2_) +0.05 M HCl hydrochloric acid with the *pH*-8 result a new disk structure morphology, is shown in Fig. 13a. The corresponding typical snapshot of fractal aggregation represents the successive electroplating of CZT thin films of growth mechanism. The disk surface micrograph of the CZT thin films has a typical minimum DLA and less fractal structure at *pH* 8 and 27 °C for 30 min. The disc CZT structure of Cu has a stick Probability (*p* = 0.01). According to the pourbaix diagram, Cu has a small sticking probability due to the higher pH of 8 and 27 °C for 30 min as per the electrochemical deposition parameter. Initially, the copper structure is found to be compact, which may be due to the screening effect of Cu(OH) and CuO at higher *pH*. The internal diffusion of Cu in Cu is less and Cu (OH) in Cu is also more found at *pH* 8. The Cu(OH) in copper is a typical diffusion to stop the growth of Cu. Hydroxide ionic species are gathered at the end of the elliptical shape causing maximum aggregates. Therefore, maximum aggregation causes less DLA with the minor fractal structure shown in the blue region in Fig. 13b. This structure is gaining support to form an elliptical disc-like structure. The second electroplating layer of Zn has a stick probability of *p* = 0.001 that is less than the first electroplating of Cu at the same electrochemical deposition parameter. The surface diffusion and diffusion coefficient of Zn in Cu are less than those of Zn(OH) and ZnO, as per the Pourbaix diagram.

The second deposition shows that the wide elliptical width of the disk and the end of the disk have more aggregation due to the screening effect of Zn (OH) and ZnO, causing less DLA. Therefore, the second electrochemical has a less fractal structure at *pH* 8 and 27 °C for 30 min than the previous *pH*-7, *pH*-6, and *pH*-5 cases shown in the green and blue regions respectively, in Fig. 13b. The interfacing diffusion of the first and second electro-chemicals are the inner and outer parts of the first blue region, as shown in Fig. 13b. The third layer of Sn electroplating on Cu–Zn has a sticking probability of *p* = 0.0005 for Sn. Sn has less sticking probability due to the higher *pH*-7 and 27 °C for 30 min as per standard and practical Pourbaix diagram as compared with previous plating as shown in the blue and green region, respectively, at the same electrochemical parameter. Sn surface diffusion in Zn is less than the interfacing of Zn–Cu thin films. The outer layer of the Sn ions does not grow as rapidly as high *pH* ions because of the screening effect of Sn(OH) and SnO. The internal diffusion of Sn in Sn is less at *pH*-8. The Sn(OH) in zinc is a typical diffusion that stops the growth of Sn. Hydroxide ionic species are gathered at the end of the disk, causing the maximum aggregates. Sn has a minimum internal diffusion of Zn–Cu. Therefore, maximum aggregation with a minor fractal structure is shown in the red region in Fig. 13b. This structure leads to the formation of an elliptical and circular-like structure of the CZT thin films. The interfacing of Cu–Zn–Sn diffusion to form an elliptical and circular structure is shown in Fig. 13b for a typical snapshot of fractal aggregation representing the successive electroplating of CZT thin films of the growth mechanism. This is consistent with the FE-SEM micrograph 4 µm scale shown in Fig. 13a.

We compared experimental and simulated fractal dimensions of approximately 1.9 and 2.0 for *pH* 8, as shown in Fig. 13c, d, respectively. The *pH*-dependent asymmetry of the experimental fractal dimension is very close to the standard fractal dimension of the simulated study.

**Figure 14:**
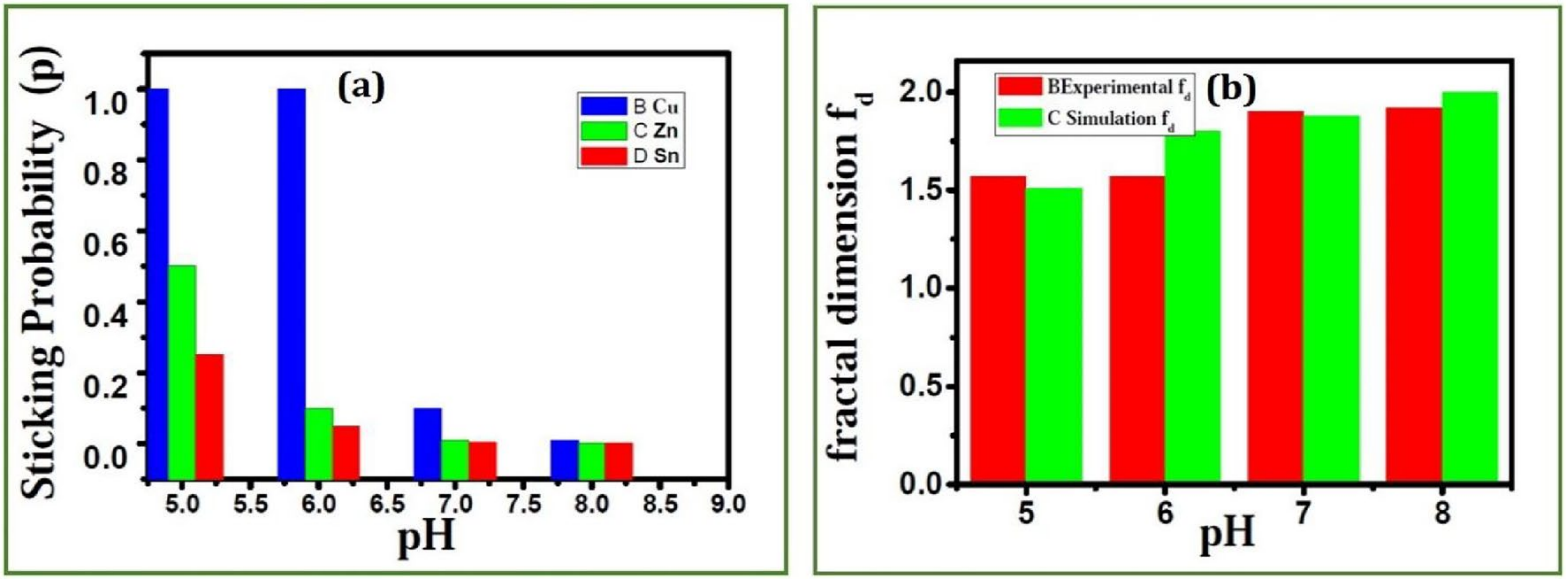
(**a**) Sticking probability simulated for the deposition of ions against pH, (**b**) Variation of fractal dimension for different *pH* values.

Figure 14a shows a comparison of the sticking probability for the deposition of different ions with different *pH* values. As described earlier, the diffusion of the ions depends on the *pH* of the solution. Both experimental and simulation results show that the fractal dimension of CZT thin films increases with *pH* (See Fig. 14b). It can be observed that the morphology of the structures changed from fractal to rod as *pH* of the solution enhances. The process is purely electrochemical and hence increasing *pH*, might have reduced the screening effect caused by outer branches thus allowing the ions to diffuse more into the structures. Such increased diffusion was previously considered by decreasing the value of sticking probability. However, in the present scenario the effect of potential which decays with distance in addition to decreased screening effect due to increase in *pH* was taken in simulation. Thus, for layered deposition, we considered the diffusion of ions might have increased in each layer which is modeled as decreasing sticking probability for Cu, Zn and Sn. At present, any possible relationship between the materials regarding the value of *p* cannot be justified. We assumed that in high *pH*, a saturation state of diffusion of ions and almost the same and independent of the layers. Thus, *p* is very low and almost the same for Cu, Zn and Sn.

When the *pH* of the solution is increased, the structure changes shape from dendrite to disk. Recently, Agasti et. al conducted a study on the co-electro deposition of Cu–Zn–Sn by varying the *pH*^42^. It was shown that electrolytes with low *pH* resulted in non-uniform rough films. When the *pH* of the electrolyte was increased, dense and smooth films were obtained. The change in morphology and irregularities are attributed to hydrogen evolution when the *pH* is low. In another study, the fractal structures were obtained using gold nanoparticles, and a morphological change from cross-like to fractal-like was observed when the particle size was increased^43^. As the particle size increases, noise fluctuation decreases, leading to fractal aggregates. We show that particle size is slightly high in low *pH* conditions, as shown in Fig. 7c, d, and this might have reduced the fluctuation due to temperature effects, leading to fractal structures with a low value of fractal dimension, as shown in Fig. 14b.

## Metal- *CZT* thin films based Schottky diodes used as top and back contacts in superstrate and substrate for solar cells

**Figure 15:**
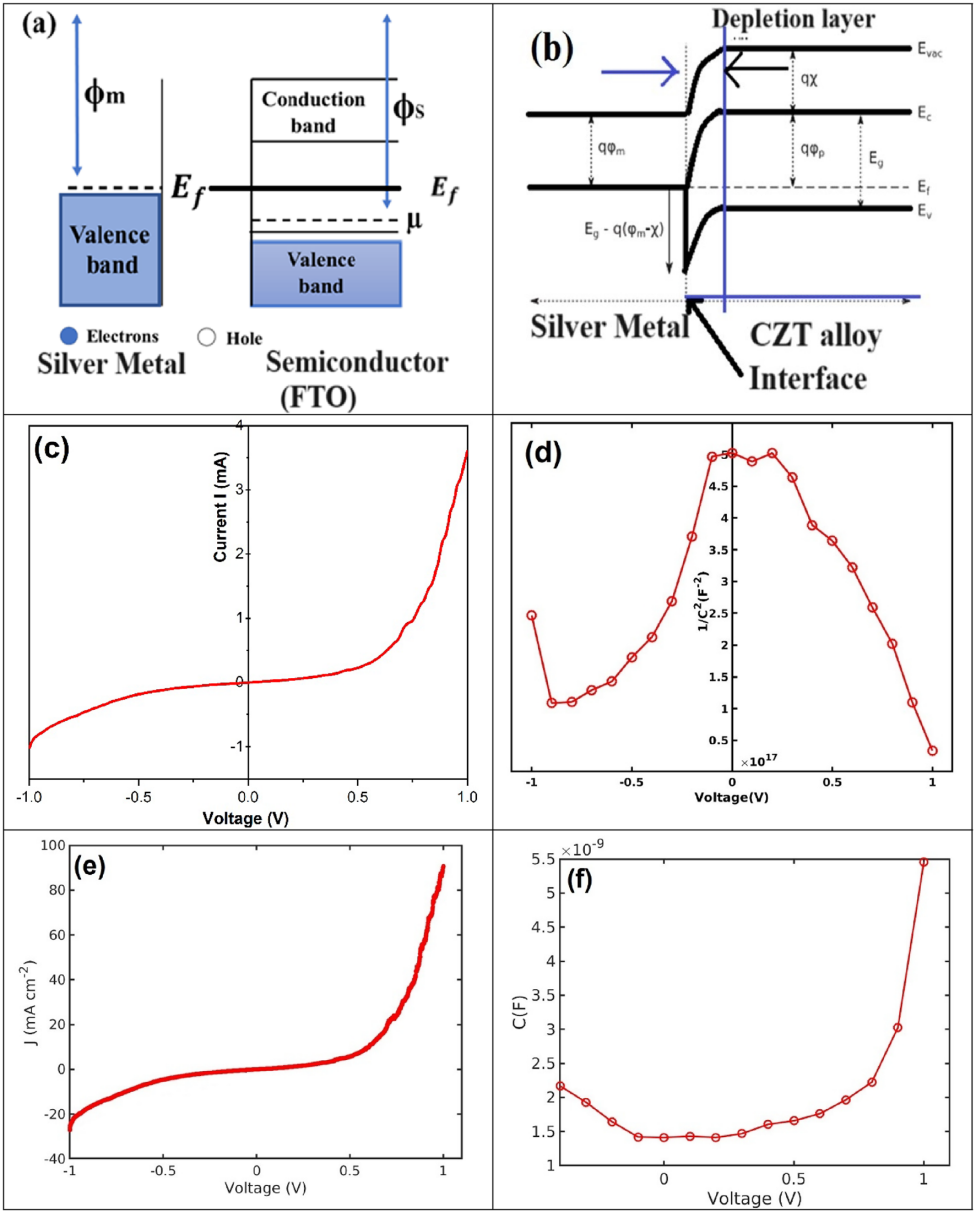
(**a**) Energy band diagram of the (Ag) metal-semiconductor (FTO) before contact, (**b**) Energy band diagram of the (Ag) metal-alloy (CZT) after contact, (**c**) Current (*I*) -Voltage (*V*) measurement, (**d**) 1/C^2^-Voltage measurement, (**e**) Current density (*J*) -Voltage (*V*) measurement, (**f**) Capacitance (*C*) -Voltage (*V*) measurement.

The current density-voltage *(J-V)* characteristics of the Schottky diodes were studied using a potentiostat device. These diodes were fabricated thoroughly using the rapid thermal evaporation technique. A typical 65 nm thin silver point of contact is formed. A metal-semiconductor interface is made by using a typical 4 mm^2^ square shadow mask. When a high vacuum up to (~ 10^−6^ Torr) range is achieved in rapid thermal evaporation an appropriate work function of 4.2 eV is observed. A high vacuum range was maintained in the diffusion vacuum pump to avoid metal oxide formation during the metal-CZT thin film interface. Generally, the researcher found that a near 100 Ampere current across the transport tungsten filament-based boat channel is required for interface design^44^. An individual energy band diagram of metal-CZT thin film interface as shown in Fig. 15a, b respectively. An energy band of metal-semiconductor (Ag-FTO) interfaces are sketched in Fig. 15a. The typical energy band diagram of metal-CZT alloy-FTO semiconductor interface constructed as shown in Fig. 15b.

Here, we have found a standard parameter for Schottky diode electrical characteristics. The electron transport mechanism of device electrical characterizations is discussed here: In these characterizations, Fig. 15c, d current-voltage (*I-V*) and capacitance-voltage (*C-V*) measurements were carried out by employing a potentiostat device (Bio-Logic, Model-SP-150) at room temperature. The EC-Lab ASCI file was used for the first I-V characterization. We have set an initial run-on channel: 1(SN-12497) proceeded, then start grouped channel (S):1 is started ON, we have used here counter electrode (CE) versus working electrode (WE) in the range of applied voltage from − 10 V to 10 V. We selected the standard electrode potential connection for diode characteristics: therefore, typical voltage ranges from minimum of −2.50 V to a maximum of 2.50 V.

In an EC-lab, Au/Pt was used as the electrode material. NaCl (0.2 M) electrolyte was used to maintain the *pH* of the buffer solution. A saturated calomel electrode (SCE) at (0.241 V) was used as a reference electrode. The standard electrode surface area was approximately 0.001 cm^2^ with a characteristic mass of 0.001 g. The equivalent weight was measured with an accuracy of 0.000 g/eq, with corresponding density with accuracy 0.000 g/cm^3^. During the Capacitance-voltage (*C-V*) measurements, a DC voltage was applied to the capacitor with a small amplitude AC voltage signal superimposed over the DC signal. Capacitance in terms of microfarad was recorded for the Schottky diode as *C-V* Schottky characteristics measurements. The *C-V* measurements were carried out at a frequency of 1 kHz as shown in Fig. 15d, f.

In Fig. 15a shows that before contact of silver metal and FTO semiconductor, φ_m_ is the metal work function of silver (Ag) and φ_s_ is work function of FTO semiconductor, 4.2 eV work function for silver, the work function φ_s_ is generally around 4.4 to 4.6 eV. E_f_ is the fermi energy of metal and semiconductor before contact. The Fermi energy of silver is typically around 5.5 eV relative to the vacuum level. The Fermi level in an n-type semiconductor like FTO is closer to the conduction band. Considering the work function of (FTO) φ_s_ is around 4.4 to 4.6 eV, and assuming the electron affinity of (FTO) χ_FTO_ is typically around 4.0 eV,

In Fig. 15b show that the work function of the silver metal (qφ_m_), is smaller than that of the CZT alloy, q is equal to the absolute value of electron charge, while, q_χ_ is electron affinity of the CZT alloy and q_φ_ is the difference between the conduction band minimum (Ec) and Fermi level of the CZT alloy. Figure 15b revels the band bending at Metal-CZT alloy Interfaces. Band bending occurs at the interface of two different materials due to the difference in their work functions and electronic properties. This is particularly relevant when discussing the interface between metals (like silver) and p-type semiconductors (like CZT alloys).

Here, we’ll outline how to understand and analyze the band bending at the interface between silver (Ag) and a CuZnSn (CZT) alloy. At equilibrium state gives band bending in p-type CZT Alloy with silver contact. Electrons will move from the material with a higher Fermi level (CZT) to the material with a lower Fermi level (Ag) until equilibrium is reached. This movement causes band bending in the semiconductor. Upward band bending: for p-type like CZT, the valence band bends upward near the interface with silver. Depletion region: A depletion region forms near the interface where the charge carriers (holes) are depleted. Electron affinity (χ): The energy needed to move an electron from the conduction band of the CZT alloy to the vacuum level.

Band bending analysis: Understanding band bending involves examining the difference in work function and electron affinity between the contacting materials. p-type CZT and Silver Contact: Results in upward band bending in the CZT near the interface with silver, forming a depletion region due to the alignment of Fermi level. For the p-type CZT alloy, this alignment causes the valence and conduction bands to bend upwards near the interface with the silver due to the higher work function of silver compared to the electron affinity of CZT.

In this situation, charges can move easily across the interface in both directions and the silver metal-CZT alloy interface is called Schottky diode. In this Fig. 15b draws a situation in which qφm is smaller than the work function of the CZT alloy. This leads to the formation of a Schottky barrier at the metal-alloy interface nature are shown in Fig. 15c, e with a barrier height of Eq. (8);

8 $$ q\varphi Bp0 = E_{g} - q\left( {\varphi_{m} - \chi } \right) $$

which stops charges from moving easily across it, like a p-n diode. This type of Schottky nature of appear in Fig. 15c, e respectively.

The current-voltage (*I-V*) and current density-voltage (*J-V*) electrical properties of the fabricated Ag-CZT thin films based on Schottky diodes were investigated using a potentiostat device in the voltage range −1 to + 1 V, as depicted in Fig. 15c–f. We found Schottky diode characteristics and parameters from this measurement, such as lower turn-on voltage (Vth), low junction capacitance *C*_*lj*_, and higher Schottky barrier height (SBH), and fast response for the Schottky junction devices, by analyzing them. The CZT thin film-based Schottky diode shows a rectifying nature with 0.5 to 0.7 V lower turn-on voltage (Vth) as shown in the Fig. 15c.

It is compared to commercial ordinary silicon diodes that show 0.6–0.7 V low turn-on voltage and germanium diodes 0.2–0.3 V for Schottky diodes. It can provide a better electron transport mechanism and enhance solar cell efficiency for the fabrication of [Ag–ZnO–ZnS–CZTS–FTO–Ag] substrate configuration of the solar cells. In this research work, we reported here that a metal-CZT thin film based Schottky diode can be used as a top and back contact in superstrate and substrate solar cells.

This fabricated Ag-CZT-based Schottky diode functions as a transitional diode between germanium and silicon at very low bias. We have measured here appropriate *J-V* measurements in the range of 20 mA/cm^2^ to 90 mA/cm^2^ and −1 to +1 V in Fig. 15e, which can give a significant interface area of 4 mm^2^. We have observed the measured interface area, which shows the rectifying electron transport mechanism in CZT thin films. This means that the current flows in one direction. The ideality factor η is the function of the applied voltage and the relation between the SBH and the depletion layer. The significant value of *V* at which the *I-V* and *J-V* rectifying characteristics acquire a Schottky nature is dependent on the parameters of the semiconductors. They can be attributed to an ideal diode. The ideal diode equation, when expressed in terms of current density (J) rather than current (*I*), is commonly used to analyze the characteristics of semiconductor diodes. The Eq. (9) is given by:

9 $$ J = ~J_{0} (e^{{\frac{{qV}}{{\eta kT}}}}  - 1) $$

where J is the current density, J_0_ is the reverse saturation current density, q is the elementary charge, V is the applied voltage, η is the ideality factor, k is the Boltzmann constant, and T is the absolute temperature. The ideality factor of Ag-CZT thin films diode is 1.786, this ideality factor calculated from ideal diode equation from the slope of the forward bias in I-V plot in the linear region. The other parameters viz. root mean square roughness (RMS in nm), point defects, line defects, surface defects, dislocation density, Schottky defects, film thickness, grain size effect, different crystal structure phases, and distinct Schottky barrier height (eV), all apparently contribute to Schottky diode performance^45,46^. At a lower turn-on voltage of 0.52–0.70 V the estimated mean error in a voltage drop of 0.61 ± 0.090 was observed in devices under forwarding bias. However, in the reverse-bias case, we have observed a reverse current density of 0.28 mA/cm^2^ devices with an estimated mean error of ±0.02 (*I-V* and *J-V* at 300 K).

These SBH devices conduct in the non-linear region under forwarding bias, but they carry more (reverse) leakage current. They do not block the leakage current across the Schottky diode in Fig. 15a.

Figure 15f show C-V plot helps in determining the doping concentration profile across the depth of the semiconductor. By applying a varying reverse bias voltage, the depletion region width changes, and the capacitance is measured as a function of this voltage. The intercept of the C-V plot can provide the built-in voltage (Vbi) of the junction, which is important for understanding the electrostatic potential across the device. The capacitance is inversely related to the depletion region width. Therefore, C-V measurements help in estimating the width of the depletion region at different applied voltages.

Figure 15d shows the slope of the 1/C^2^ versus V plot can be used to extract the doping concentration (N*d*) of the semiconductor. For a uniformly doped material, the plot is linear, and the slope is inversely proportional to the doping concentration. Extrapolating the linear region of the 1/C^2^-V plot to the voltage axis provides the built-in potential (Vbi). This value is crucial for understanding the junction properties and potential barriers within the device. Deviations from linearity in the 1/C^2^-V plot can indicate the presence of interface states, traps, or other defects. These defects can affect device performance by introducing recombination centers or trapping sites.

## Conclusions

We have extensively carried out experimental and simulation work to obtain different surface morphologies of CZT thin films deposited on an FTO substrate using the electrochemical deposition method. XRD studies confirmed the formation of kesterite structures of CZT thin films. We have changed the *pH* of the solution, which shows the morphological transformation from fern-like dendrite to disk structure with increasing *pH* of the solution. This was further examined using the fractal dimension of the aggregates. The fractal dimension of the aggregates decreased with increasing *pH* of the solution. The screening effect increases during the formation of disks, which leads to a compact structure with a high fractal dimension.

The morphological evolution of the CZT thin films is further carefully examined through DLA simulations. It is found that the initial deposition of Cu plays an important role in forming the global structure. Different *pH* conditions were successfully simulated by changing the sticking probability from 0.0005–1.0 for diffusing ions. There is a clear *pH*-dependent morphological change from dendrite to disk obtained in simulations, similar to the case of experiments. Further, we calculated fractal dimensions using the box-counting method and found that as *pH* increased from 5 to 8, it increased from 1.5 to 2.0. The experimental and theoretical study was conducted for the first time, yielding a quantitative description of the microstructure evolution of CZT thin film growth. The electrical characteristics and parameters of the Schottky diodes based on fabricated Ag-CZT thin films were determined through current-voltage (*I-V*) and current density-voltage (*J-V*) measurements. The Schottky diodes, which are based on the Ag-CZT thin films, exhibit an improved electron transport mechanism that enhances the efficiency of solar cells.

## Data Availability

The data presented in this study are available upon request from the corresponding author.
